# Radiation induces NORAD expression to promote ESCC radiotherapy resistance via EEPD1/ATR/Chk1 signalling and by inhibiting pri-miR-199a1 processing and the exosomal transfer of miR-199a-5p

**DOI:** 10.1186/s13046-021-02084-5

**Published:** 2021-09-29

**Authors:** Yuchen Sun, Jizhao Wang, Yuan Ma, Jing Li, Xuanzi Sun, Xu Zhao, Xiaobo Shi, Yunfeng Hu, Fengyi Qu, Xiaozhi Zhang

**Affiliations:** 1 The Department of Radiation Oncology, The First Affiliated Hospital of Xi’an Jiaotong University, 277 West Yanta Road, Xi’an, Shaanxi 710061 People’s Republic of China; 2 The Department of Thoracic Surgery, The First Affiliated Hospital of Xi’an Jiaotong University, 277 West Yanta Road, Xi’an, Shaanxi 710061 People’s Republic of China; 3 The Department of Radiation Oncology, The Second Affiliated Hospital of Xi’an Jiaotong University, 157 XiWu Road, Xi’an, Shaanxi 710004 People’s Republic of China; 4 The Department of Radiation Oncology, Yanan University Affiliated Hospital, 157 Beida Road, Yanan, Shannxi 716099 People’s Republic of China

**Keywords:** Esophageal squamous cell carcinoma, Radioresistance, NORAD, Pri-miR-199a1, EEPD1

## Abstract

**Background:**

Radioresistance, a poorly understood phenomenon, results in the failure of radiotherapy and subsequent local recurrence, threatening a large proportion of patients with ESCC. To date, lncRNAs have been reported to be involved in diverse biological processes, including radioresistance.

**Methods:**

FISH and qRT–PCR were adopted to examine the expression and localization of lncRNA-NORAD, pri-miR-199a1 and miR-199a-5p. Electron microscopy and nanoparticle tracking analysis (NTA) were conducted to observe and identify exosomes. High-throughput microRNAs sequencing and TMT mass spectrometry were performed to identify the functional miRNA and proteins. A series of in vitro and in vivo experiments were performed to investigate the biological effect of NORAD. ChIP, RIP-qPCR, co-IP and dual-luciferase reporter assays were conducted to explore the interaction of related RNAs and proteins.

**Results:**

We show here that DNA damage activates the noncoding RNA NORAD, which is critical for ESCC radioresistance. NORAD was expressed at high levels in radioresistant ESCC cells. Radiation treatment promotes NORAD expression by enhancing H3K4me2 enrichment in its sequence. NORAD knockdown cells exhibit significant hypersensitivity to radiation in vivo *and* in vitro. NORAD is required to initiate the repair and restart of stalled forks, G2 cycle arrest and homologous recombination repair upon radiation treatment. Mechanistically, NORAD inhibits miR-199a-5p expression by competitively binding PUM1 from pri-miR-199a1, inhibiting the processing of pri-miR-199a1. Mature miR-199a-5p in NORAD knockdown cells is packaged into exosomes; miR-199a-5p restores the radiosensitivity of radioresistant cells by targeting EEPD1 and then inhibiting the ATR/Chk1 signalling pathway. Simultaneously, NORAD knockdown inhibits the ubiquitination of PD-L1, leading to a better response to radiation and anti-PD-1 treatment in a mouse model.

**Conclusions:**

Based on the findings of this study, lncRNA-NORAD represents a potential treatment target for improving the efficiency of immunotherapy in combination with radiation in ESCC.

**Supplementary Information:**

The online version contains supplementary material available at 10.1186/s13046-021-02084-5.

## Background

Esophageal cancer, one of the most lethal malignancies worldwide [1], is mainly histologically classified into esophageal adenocarcinoma (EAC) and esophageal squamous cell carcinoma (ESCC). In Asian countries, ESCC is the most common histological type and has a worse prognosis than EAC. Although surgery is the most effective treatment for prolonging survival, a large number of patients are not candidates for direct esophagectomy [2–4]. Comprehensive therapy, including radiation and chemotherapy, is introduced to either improve the effects of surgery or control the growth of cancer lesions in patients with nonresectable tumours. Radiation is an important part of combined therapy, representing an efficient method to control local recurrence and optimize surgical strategies. For example, preoperative chemotherapy combined with radiotherapy is required for cervical esophageal cancer [5]. Both definitive and preoperative radiation are important therapeutic strategies that prolong the overall survival of patients with ESCC [6]. Although chemoradiotherapy tremendously reduces the local recurrence rate, approximately 40–60% of patients still experience local recurrence after concurrent chemoradiotherapy [4, 7], partially due to radiation or chemotherapy resistance. Although substantial progress has been achieved in identifying novel biomarkers and therapeutic targets for improving radiation sensitivity, the molecular mechanism underlying radioresistance remains ambiguous and complex; it is proposed to involve cell cycle checkpoints that prevent cancer cells from sustaining radiation-induced DNA damage, activation of the DNA damage response, the self-renewal of cancer stem cells, the epithelial-mesenchymal transition (EMT), etc. [7, 8] In this manuscript, we aimed to identify key genes that participate in sensitizing ESCC cells to radiation therapy and to lay the foundation for drug development.

In recent years, long noncoding RNAs (lncRNAs) have attracted increasing attention as a result of their diverse biological functions in the development and progression of multiple cancers [9]. Combined with our previous studies, the DNA damage-activated noncoding RNA-NORAD (also named linc00657) garnered our attention. NORAD is abundantly expressed in multiple eukaryotic cells, is conserved across mammalian species and has been shown to function as an oncogene in numerous cancers. NORAD maintains genomic stability by sequestering and negatively regulating PUMILIO proteins through its 18 conserved PUMILIO response elements [10]. Another study reported that NORAD interacts with DNA damage repair- and DNA replication-related proteins, assembling a topoisomerase complex to sustain genomic stability in response to DNA damage [11]. In addition, NORAD binds to a group of microRNAs, modulating their abundance to exert oncogenic functions [12, 13]. In our previous study, we identified that NORAD knockdown significantly sensitized ESCC cells to radiation [14]. However, the mechanism by which NORAD regulates radiobiological processes in ESCC is unclear.

The main anticancer mechanism of radiation is the generation of a cluster of lethal lesions, which in turn induce DNA damage in cells and tissues [15]. Damaged DNA is repaired by the activation of homologous recombination (HR) and nonhomologous end joining (NHEJ) pathways in vitro [16]. The crosstalk between DNA damage repair and radiosensitivity is obvious [17]; It is notable that some studies have found that inhibiting DNA damage repair increases the efficiency of immune checkpoint therapy, especially when combined with radiotherapy. Frank P et al. reported that the ATR kinase inhibitor AZD6738 reduces the exhaustion of CD8+ T cells induced by radiation. In addition, AZD6738 combined with radiation generates immunologic memory for tumours in mice [18]. According to a recent study, DNA double-strand breaks promote PD-L1 expression by activating STAT1 and STAT3 signalling [19]. TMT-based proteomics analyses also suggest the potential function of NORAD in “Response to stimulus” and “Immune system process”. However, the mechanism by which suppressed DNA damage repair responses increase the efficiency of immune checkpoint therapy in combination with radiotherapy remains elusive. In this context, we hypothesize that NORAD modulates ESCC radiosensitivity by regulating the DNA damage repair process. We assume that NORAD is involved in the synergy between radiotherapy and immune checkpoint therapy.

## Methods

### Cell culture, lentivirus transfection and plasmid transduction

The esophageal squamous carcinoma cell lines KYSE-150 and TE-1 were purchased from the Cell Bank of the Chinese Academy of Sciences Typical Culture Preservation Committee (Shanghai, China). Cells were maintained in Roswell Park Memorial Institute (RPMI) 1640 medium supplemented with 10% FBS and 1% penicillin/streptomycin. Cells were cultured in a 5% CO_2_ incubator at 37 °C. Radioresistant cells were generated from parental KYSE-150 and TE-1 cells by seeding the cells into 6-well plates and then exposing them to radiation at a dosage of 2 Gy per day for 30 days. The surviving cells were characterized as radioresistant cells (termed KYSE-150R and TE-1R cells). The generation of radioresistant cells was validated by performing colony formation assays.

The sh-NORAD lentivirus (*H. sapiens*), sh-NORAD lentivirus (*M. musculus*), sh-EEPD1 and their corresponding control lentiviruses were purchased from GeneChem (Shanghai, China). The miR-199a-5p mimics, miR-199a-5p inhibitors, siRNA against PUM1 and EEPD1 overexpression vector were purchased from GenePharma (Shanghai, China). The PUM1 overexpression vector and PUM1-ΔHD overexpression vector were purchased from GeneChem (Shanghai, China). For virus infection, KYSE-150 and TE-1 cells were seeded into 48-well plates and grown to 50% confluence. Lentivirus (1 × 10^8^ TU/ml) and infection reagents were mixed and added to the cells. The medium was changed after 48 h of infection, and puromycin (3 μg/ml) was added to the medium to select cells that were successfully infected. Transient knockdown or overexpression of candidate genes was achieved by transfecting the appropriate siRNAs or gene overexpression plasmids. Transient transfections were performed using Lipofectamine 3000 (Invitrogen/Thermo Fisher Scientific) according to a standard protocol.

### Patients cohorts

Two clinical cohorts of esophageal squamous cell carcinoma were involved in the study. Firstly, based on TCGA datasets, clinical data and RNA-sequence data of ESCC patients with radiotherapy response information were downloaded (https://www.cancer.gov/about-nci/organization/ccg/research/structural genomics/tcga). The inclusion criteria from TCGA datasets were as follows:(1) patients were histologically diagnosed with ESCC; (2) patients had complete gene expression profile data; (3) patients completed the follow up; (4) Patients with clearly information of radiotherapy response, including complete response, partial response, stable disease and radiographic progressive disease.

Secondly, we further enrolled an independent cohort of 77 ESCC patients who underwent esophagectomy and adjuvant chemo-radiotherapy in the First Affiliated Hospital of Xi’an JiaoTong University. Tissues were collected during surgery and were used for FISH and IHC examination. Patients with local recurrence in 12 months after radiotherapy completion were recruited into chemoradiotherapy resistant group; and other patients were assigned into chemoradiotherapy sensitive group. The study was approved by the Ethics Committee of The First Affiliated Hospital of Xi’an Jiao Tong University.

### qRT–PCR

Total RNA was extracted from KYSE-150 and TE-1 cells using TRIzol reagent (Invitrogen, Carlsbad, CA, USA) and then reverse transcribed into cDNAs using a PrimeScript™ RT reagent kit (TaKaRa, Japan). MicroRNA-specific cDNAs were generated using a Mir-X™ miRNA First Strand Synthesis kit (TaKaRa, Japan). qRT–PCR was carried out using SYBR Premix Ex Taq™ II (TaKaRa, Japan). GAPDH and U6 were used as reference genes for mRNA and microRNA expression, respectively.

### Western blot

Total protein was extracted from KYSE-150 and TE-1 cells using RIPA buffer (Sigma Aldrich, Cambridge, MA) and quantified using a BCA kit (Sigma Aldrich, Cambridge, MA). Then, proteins were separated on 10% SDS–PAGE gels and transferred to PVDF membranes, which were then incubated with primary antibodies at 4 °C overnight. Secondary antibodies were then incubated with the membranes for 1 h at room temperature before the protein bands were visualized using an ECL kit.

### Extraction and identification of exosomes

Electron microscopy was used to observe and identify the extracellular vesicle-like structure of exosomes. Nanoparticle tracking analysis (NTA) was conducted to test the particle size-concentration distribution using a ZetaView PMX 110 instrument (Particel Metrix, Germany). CD63 was considered an exosome marker, and protein expression was evaluated using western blotting. For the exosome tracer experiment, 2000 cells were seeded in each well of a 96-well plate; the exosome suspension was incubated with 2 μM PKH26 for 5 min at room temperature and added to the target cells in a volume of 20 μl/well. Red fluorescence indicates the process of exosomes entering target cells.

### Fluorescence in situ hybridization

We used KYSE-150 and TE-1 cells to produce slides that were pretreated with HCl and then fixed with neutral formalin. A probe targeting NORAD was used for hybridization with cell slides after treatment with the diluted prehybridization solution. DAPI was used to stain nuclei, and cell slides were observed using confocal microscopy.

### Immunofluorescence staining

KYSE-150 and TE-1 cells were seeded on glass slides in 48-well plates and cultured overnight. Cells on the slides were fixed with 4% paraformaldehyde before they were treated with Triton X-100 (5%) to permeabilize cells and a BSA solution to block nonspecific binding. Cells were treated with primary antibodies and incubated at 4 °C overnight. After the unbound primary antibody was removed, the cells were then incubated with PE-conjugated secondary antibody for 1 h at 37 °C. DAPI was added to the slides for nuclear staining, and glycerin was used to block the slides. Images of the cells were obtained under a confocal microscope. The primary antibodies and PE-conjugated secondary antibody were purchased from Abcam.

### Co-IP

Cells were lysed at 4 °C for 5 min in RIPA buffer containing protease inhibitors. Whole-cell lysates were then precleared with Protein A/G beads. The PD-L1 antibody was then added and incubated overnight at 4 °C. The antibody/antigen complex was pulled down from the lysates by using Protein A/G-coupled agarose beads. Beads were washed with RIPA buffer 3 times and resuspended in loading buffer. Western blot assays were described previously.

### RNA immunoprecipitation (RIP)

We purchased a Magna RIP™ RNA-binding Protein Immunoprecipitation Kit (Millipore, Billerica, MA, USA) to analyse the interaction between RNA and proteins. KYSE-150 cells were lysed in RIP lysis buffer for further experiments. We used an IgG antibody as the negative control. The expression levels of NORAD and pri-miR-199a1 were evaluated using qRT–PCR.

### Homologous recombination (HR) reporter assay

HR reporter and I-SceI expression plasmids were purchased from GeneChem (Shanghai, China). For the HR reporter assay, KYSE-150-sh-NC and KYSE-150-sh-NORAD cells were transfected with the HR reporter. G418 (1 mg/ml) was used to select successfully transfected cells. Then, these cells were transfected with the I-Scel plasmid to induce double-strand breaks in the HR reporter plasmid. Cells were harvested for analysis after 48 h. Cells that underwent HR repair were positive for green fluorescence.

### Apoptosis and cell cycle analyses

We used an Annexin V-APC/7-AAD Apoptosis Detection Kit I (BD Pharmingen™, New Jersey, USA) for the apoptosis assay. NORAD knockdown and NC cells were harvested and resuspended (5 × 10^5^ cells per sample) before 5 μl of Annexin V-APC and 7-AAD were added to the suspensions and incubated for 15 min at room temperature in the dark. Flow cytometry was used to evaluate the luciferase intensity of Annexin V-APC and 7-AAD. A Cell Cycle Staining Kit (BD Pharmingen™, New Jersey, USA) was used to evaluate the cell cycle distribution. Cells were fixed with 70% ethanol for 2 h at 4 °C. Target cells were treated with the DNA staining solution and permeabilization solution in the dark for 15 min at room temperature. Flow cytometry was used to evaluate the luciferase intensity at 24 h after treatment.

### Replication fork recovery

Immunofluorescence assays of BrdU foci (BrdU in double-stranded DNA) were used to evaluate replication fork recovery after exposing ESCC cells to radiation [20, 21]. BrdU was incorporated into double-stranded DNA, and the immunofluorescence intensity of BrdU foci represented the activation of replication fork recovery. Cells were exposed to 8 Gy of radiation before BrdU was added to the culture medium at a final concentration of 10 μM and incubated with the cells for 30 min. Cells with active replication forks were the cells with positive BrdU foci under a fluorescence microscope. Cells that did not receive radiation treatment were used as the control group.

### Colony formation assay and X-ray radiation treatment

Cells (500, 1000, 2000, 4000, or 8000 per well) were seeded into 6-well plates and cultured overnight in a 5% CO_2_ incubator before they were exposed to 0, 2, 4, 6, or 8 Gy of X-ray radiation. Colonies containing more than 50 cells were counted after 2 weeks of incubation. The survival curve obtained after radiation was fitted based on the single-hit multitarget model: SF = 1-(1-e ^(−kD)^)^N^. Cells were irradiated with 0, 2, 4, 6, and 8 Gy of 6 MV X-ray radiation using an X-ray linear accelerator (Clinac 2100EX, Varian Medical Systems) with a 200 cGy/min dose rate, 20 cm × 20 cm field size (FS) and 100 cm (Source-Surfaced Distance, SSD).

### Tandem mass tag proteomic-based quantitative proteome analysis

Cells successfully infected with lentivirus containing sh-NORAD or sh-NC were sonicated in lysis buffer (8 M urea and 1% protease inhibitor cocktail) to extract total protein. The protein sample was pretreated with dithiothreitol, alkylated with iodoacetamide and diluted with TEAB. Then, the protein mixture was incubated with trypsin, desalted on a Strata X C18 SPE column (Phenomenex) and vacuum-dried. A tandem mass tag (TMT) kit was used for further labelling. The peptides were subsequently evaluated using tandem mass spectrometry (MS/MS) on a Q Exactive™ Plus (Thermo) coupled to the UHPLC system. The data-dependent procedure alternated between 1 MS scan followed by 20 MS/MS scans with 15.0 s dynamic exclusion and an automatic gain control (AGC) of 5E4. The fixed first mass was set to 100 m/z.

### Xenograft mouse model and radiation treatment

ESCC cells suspended in phosphate-buffered saline were mixed with Matrigel and injected into the right flanks of nude mice (KYSE-150 model, 5 × 10^6^ cells) and C57BL/6 mice (AKR model, 1 × 10^6^ cells). After the tumour volume reached 50–100 mm^3^ in the KYSE-150 model and 150 mm^3^ in the AKR model, local radiation (2 Gy/day for 4 consecutive days) was administered to the tumour site. Mice receiving concurrent treatment were also injected with the anti-PD-1 antibody at 0, 7, and 14 days after the first radiation treatment. The tumour volume was measured with callipers and calculated using the following formula: tumour volume = 0.5× width^2^ × length.

### Whole exome sequencing

The Agilent V6 Exon + UTR probe was used. Raw paired-end reads were screened to generate clean reads. Then, BWA was used to compare the clean reads to H19 and rearrange the clean reads according to karyotype with Picard. Then, Samtools and Picard were used to remove redundant reads. Afterwards, GATK was used to generate the SNP/INDEL variant information, which was annotated by VEP. The tumour mutation burden (TMB) was then calculated (TMB = total somatic mutations/total length of target region).

### Statistical analyses

GraphPad Prism 8.2.1 and R 3.3.1 software were used for statistical analyses and data visualization. Normally distributed continuous variables, including qRT–PCR results and cell survival fraction results, were compared using independent-samples T tests. The data are presented as the means ± standard errors of the means (SEM). Abnormally distributed continuous variables are reported as medians (interquartile ranges), and categorical variables are reported as numbers (percentages). The Mann–Whitney U test was used to compare categorical variables and abnormally distributed variables between two groups. One-way ANOVA was used to compare multiple groups. Correlations between NORAD and several H3K4 methyltransferases were examined by calculating Pearson’s correlation coefficients. The χ^2^ test was used to compare the differences in clinical characteristics between different groups. *P* < 0.05 was considered statistically significant.

## Results

### High NORAD expression indicates ESCC radioresistance

**Fig. 1:**
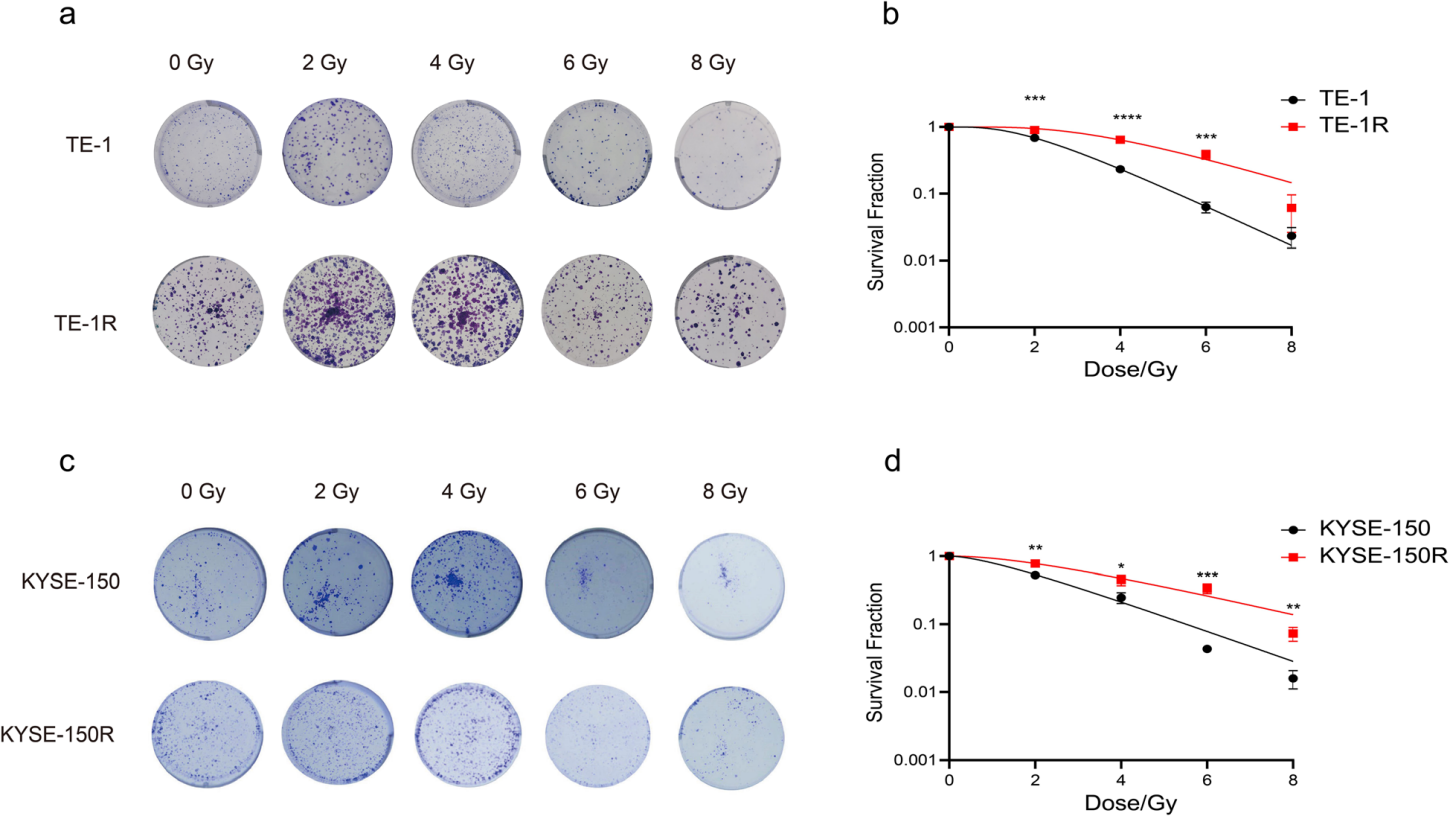
**a** TE-1 and TE-1R were exposed to 0, 2, 4, 6, 8 Gy of X-rays. Colonies were stained and pictured after 14 days. **b** Nonlinear regression(curve fit) of TE-1 and TE-1R survival fraction at different doses was curved based on the formula of Survival Fraction = 1-(1-e^-kD^)^N^. **c-d**. **c** KYSE-150 and KYSE-150R were exposed to 0, 2, 4, 6, 8 Gy of X-rays. Colonies were stained and pictured after 14 days. **d** Nonlinear regression(curve fit) of KYSE-150 and KYSE-150R survival fraction at different doses was curved based on the formula of Survival Fraction = 1-(1-e^-kD^)^N^*. (*P* < 0.05, **P < 0.01, *** *P* < 0.001, ****P < 0.0001)

**Fig. 2:**
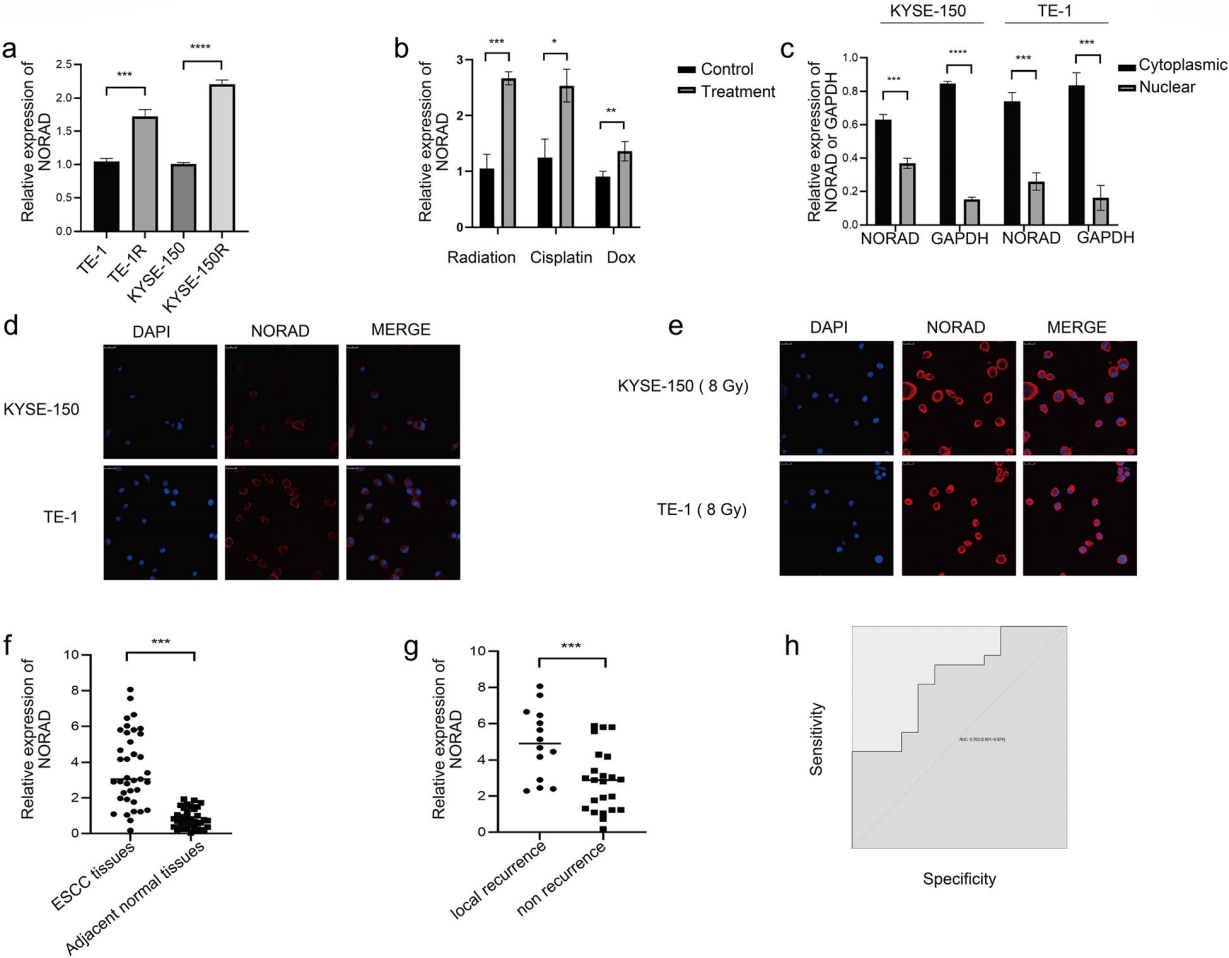
**a** qRT–PCR analysis of the expression of NORAD in radioresistant ESCC cells (KYSE-150R and TE-1R) and their parental cells (KYSE-150 and TE-1). **b** qRT–PCR analysis of NORAD expression in KYSE-150 cells treated with 8 Gy of X-ray radiation, cisplatin or doxorubicin. **c** Subcellular fractionation followed by qRT–PCR analysis of NORAD expression in KYSE-150 and TE-1 cells (GAPDH was used as a cytoplasmic control). **d-e** FISH results for NORAD expression in KYSE-150 and TE-1 cells before (**d**) and after (**e**) 8 Gy X-ray treatment. The scale bar represents 25 μm. **f** qRT–PCR measurement of NORAD expression in ESCC tissues and adjacent normal tissues. **g** qRT–PCR measurement of NORAD expression in ESCC tissues from patients with local recurrence and nonrecurrence. **h** ROC curve for predicting ESCC local recurrence based on NORAD expression. AUC = 0.763 (0.601–0.924; *p* < 0.05). (qRT–PCR results were quantified relative to GAPDH, *n* = 3 technical replicates, *P < 0.05, **P < 0.01, *** *P* < 0.001, ****P < 0.0001)

We established the acquired radioresistant ESCC cells (termed KYSE-150R and TE-1R) using the protocol described in Method section to explore the mechanism of NORAD in ESCC radioresistance. The results of the cell colony formation assay and survival fractions of TE-1, TE-1R (Fig. 1a-b) and KYSE-150, KYSE-150R (Fig. 1c-d)are presented. Survival fractions of KYSE-150 and TE-1 were significantly lower than KYSE-150R and TE-1R respectively upon radiation treatment. The results validated that KYSE-150R and TE-1R cells presented radioresistance characteristics compared with KYSE-150 and TE-1 cells, respectively. The qRT–PCR results showed that NORAD expression was upregulated in KYSE-150R and TE-1R cells compared with KYSE-150 and TE-1 cells (Fig. 2a). Following 8 Gy X-ray radiation and the administration of other genotoxic agents (doxorubicin and cisplatin), NORAD expression was approximately 2–3-fold higher in KYSE-150 cells (Fig. 2b). qRT–PCR and FISH results confirmed the significant increase in cytoplasmic NORAD expression but not nuclear NORAD expression in response to radiation (Fig. 2c-e). FISH results from 77 patients with ESCC who had been treated with radiotherapy indicated that NORAD was mainly located in the cytoplasm (Supplementary Fig. 1A). Thus, NORAD expression was subsequently examined in 37 ESCC tissues using qRT–PCR (Fig. 2f). High NORAD expression was associated with local recurrence after radiotherapy in patients with ESCC (Fig. 2g). ROC curves indicated that NORAD strongly predicted local recurrence in patients with ESCC who were treated with radiotherapy (Fig. 2h).

### Radiation activates NORAD expression by enhancing H3K4me2 enrichment

**Fig. 3:**
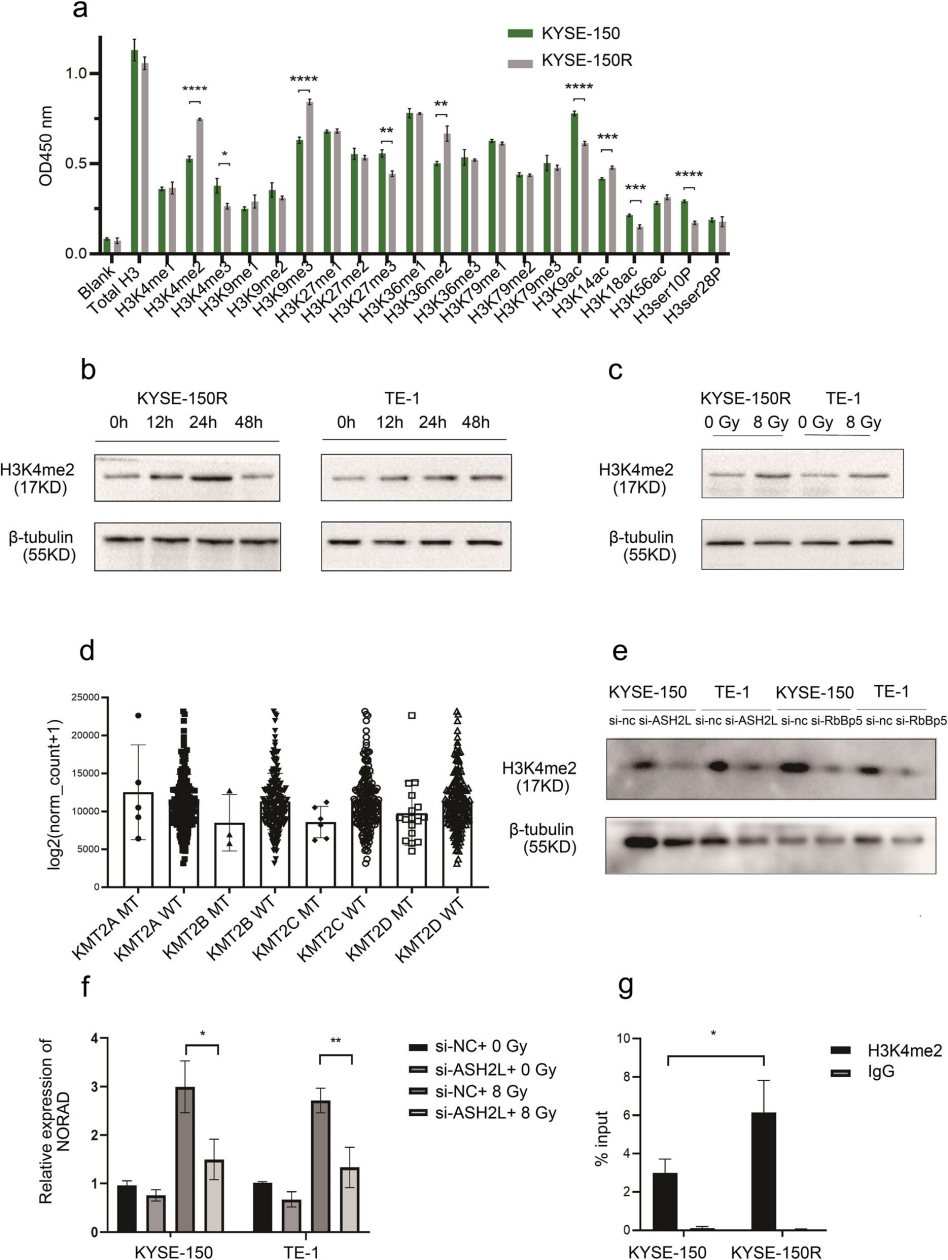
**a** OD450 values of several H3 histone proteins in KYSE-150R and KYSE-150 cells. One hundred nanograms of total histone proteins per well were used. **b** The level of H3K4me2 measured in KYSE-150 and TE-1 cells at 0, 12, 24, and 48 h post 8 Gy X-ray radiation treatment. **c** The level of H3K4me2 measured in KYSE-150 and TE-1 cells at 24 h after 8 Gy X-ray radiation or 0 Gy X-ray treatment. **d** The expression level of NORAD in patients with ESCC presenting KMT2A, KMT2B, KMT2C and KMT2D mutant type (MT) or wild type (WT) based on TCGA datasets. **e** The expression level of H3K4me2 measured in KYSE-150 and TE-1 cells with or without ASH2L and RbBP5 knockdown. **f** qRT-PCR measurement of the NROAD expression in KYSE-150 and TE-1 cells with or without ASH2L knockdown, which were treated with 8 Gy X-rays treatment. **g** ChIP-qPCR analysis of NORAD and H3K4me2 were performed in KYSE-150 and KYSE-150R cells and immunoprecipitated using H3K4me2; IgG was taken as a negative control. (qRT-PCR results quantification relative to GAPDH, *n* = 3 technical replicates, *P < 0.05, **P < 0.01, *** *P* < 0.001, ****P < 0.0001)

We next measured histone H3 expression in radioresistant ESCC cells and their parental cells to explore how radiation treatment increased NORAD expression. That the levels of H3K4me2, H3K4me3, H3K9me3, H3K9ac and other histone proteins were changed in KYSE-150R cells compared with KYSE-150 cells (Fig. 3a). We next searched the UCSC database for histone enrichment in the NORAD region in multiple cancer cells (http://genome.ucsc.edu/) and found that the NORAD sequence showed high enrichment of H3K4me2, H3K4me3, H3K9ac, H3K14ac and H3K18ac (Supplementary Fig. 1B). Western blot analysis showed a dynamic change in H3K4me2 levels after 8 Gy irradiation treatment, reaching a peak at 24 h (Fig. 3b). Compared with the control group, the level of H3K4me2 was increased in the 8 Gy radiation treatment group (detected at 24 h post irradiation) (Fig. 3c). We next analysed the correlation between NORAD expression and each H3K4 methyltransferase in SET1/COMPASS, including six homologues (SET1A, SET1B, KMT2A, KMT2B, KMT2C and KMT2D) and four common subunits, WRAD (WDR5, RbBP5, ASH2L and DPY30), based on TCGA datasets. NORAD expression was positively correlated with the expression of most H3K4 methyltransferases (Supplementary Fig. 2). Furthermore, based on TCGA datasets, patients with ESCC carrying KMT2B, KMT2C or KMT2D mutations showed slightly lower NORAD expression, but the difference was not significant (Fig. 3d). SET1/COMPASS subunits such as MLL1 alone show weak H3K4 methylation activity, complex formation with WRAD allows it to predominantly methylate H3K4 [22]. Expression of the WRAD subunits ASH2L and RbBP5 was positively correlated with NORAD expression in esophageal carcinoma tissues. We next knocked down ASH2L and RbBP5 in KYSE-150 cells. Knockdown of ASH2L decreased H3K4me2 levels (Fig. 3e, Supplementary Fig. 1C). Furthermore, ASH2L knockdown reduced NORAD expression and impaired the upregulation of NORAD upon radiation (Fig. 3f). We next performed a ChIP assay and found that H3K4me2 was enriched in the NORAD region. The intensity of H3K4me2 enrichment was increased in radioresistant cells compared with parental cells (Fig. 3g). Taken together, the radiation-induced increase in NORAD expression was due to enhanced H3K4me2 enrichment at the NORAD region.

### NORAD knockdown inhibits the replication fork restart and promotes cell apoptosis upon radiation stress

**Fig. 4:**
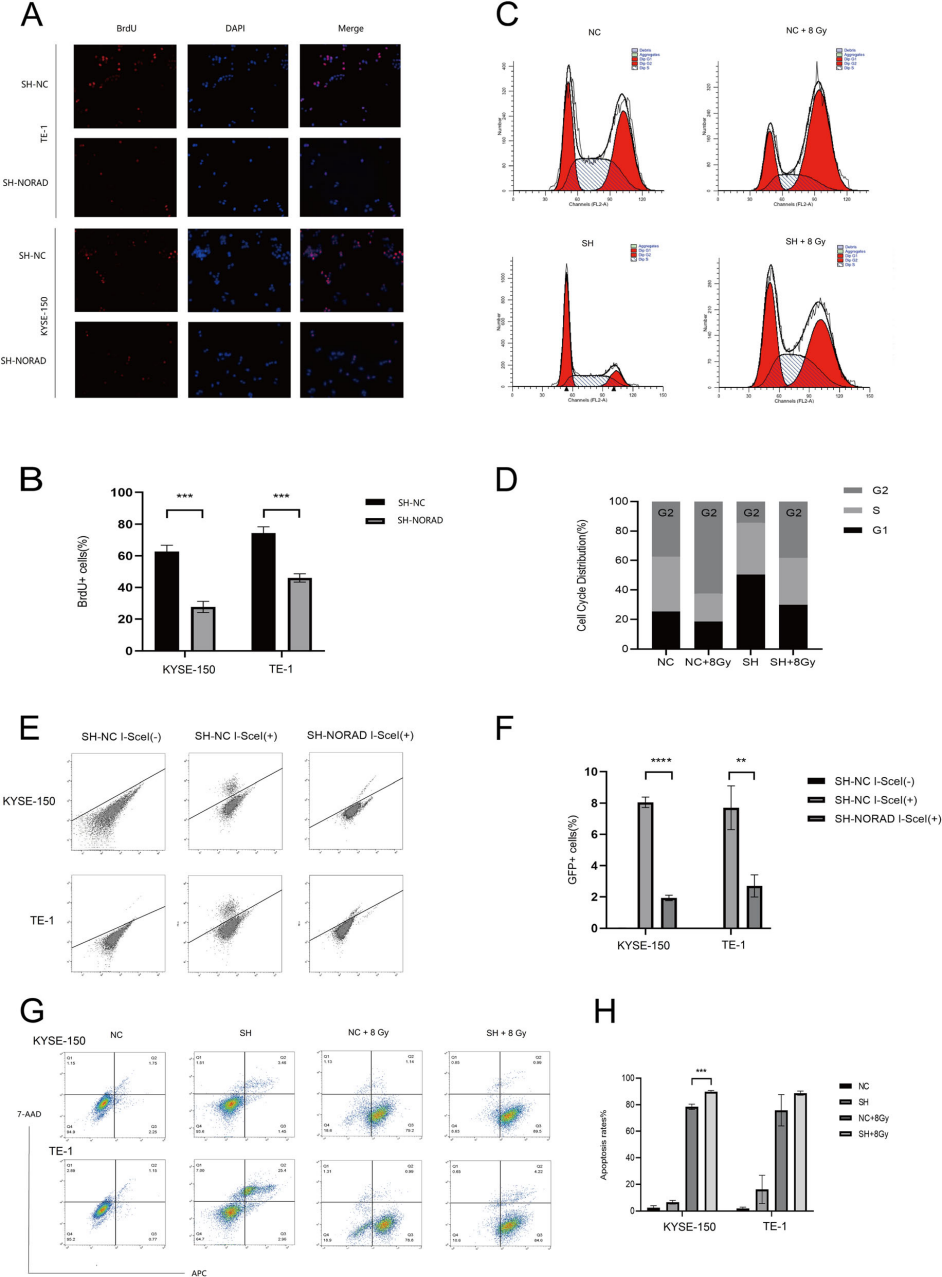
**a** Replication fork recovery was assayed as the percentage of cells with positive BrdU foci at 30 min after 8 Gy X-ray treatment. Representative immunofluorescence images are shown for TE-1 and KYSE-150 cells transfected with SH-NORAD or SH-NC. Image magnification (400×). **b** The percentage of BrdU-positive cells was quantified and analysed. **c** The cell cycle distribution of KYSE-150 cells transfected with SH-NORAD or SH-NC was measured using flow cytometry. Cells were exposed to 8 Gy of X-ray radiation or mock treatment. **d** The percentage of cells in each cell cycle phase in Fig. 4(**c**) is shown. **e** KYSE-150 and TE-1 cells were transduced with an I-SceI expression plasmid to induce DSBs, and homologous recombination repair in NORAD knockdown cells and control cells was assayed using flow cytometry and presented as the percentage of GFP-positive cells. **f** The GFP-positive percentage of each cell was quantified and analysed. **g** Cell apoptosis rates in KYSE-150 and TE-1 cells transfected with SH-NC or SH-NORAD and exposed to 8 Gy radiation or mock treatment were measured using flow cytometry. **h** The cell apoptosis rates were analysed. (*n* = 3 technical replicates, *P < 0.05, **P < 0.01, *** *P* < 0.001, ****P < 0.0001)

We next knocked down NORAD in KYSE-150 and TE-1 cells to investigate how NORAD affected the DNA damage repair response of ESCC cells during radiation stress, and the cells were designated SH-NORAD-KYSE-150 and SH-NORAD-TE-1, respectively. BrdU immunofluorescence assays were adopted to measure the replication fork restart after replication stalling upon DNA damage. Nascent DNA after DNA damage was labelled with BrdU. As observed under the fluorescence microscope, fewer BrdU-positive cells were observed in the NORAD knockdown group, suggesting that NORAD knockdown delayed the recovery of the stalled replication fork after radiation treatment (Fig. 4a-b). Regarding the cell cycle distribution, NORAD knockdown arrested ESCC cells at G1 phase. We exposed ESCC cells to 8 Gy radiation and found that radiation treatment caused arrest in G2 phase, whereas NORAD knockdown attenuated the G2 phase arrest of cells upon radiation treatment (Fig. 4c-d). Recombinant DR-GFP plasmids were transfected into cells with and without NORAD knockdown to determine whether NORAD regulated DNA repair. DNA double-strand breaks were induced by transiently transfecting the I-SceI plasmid into cells. Flow cytometry assays revealed that NORAD knockdown led to a 3–4-fold reduction in homologous recombination repair upon DSBs (Fig. 4e-f). Flow cytometry-based apoptosis assays indicated that NORAD knockdown increased the apoptosis rates of irradiated cells (Fig. 4g-h).

### Exosomes derived from NORAD knockdown cells sensitize ESCC cells to irradiation

**Fig. 5:**
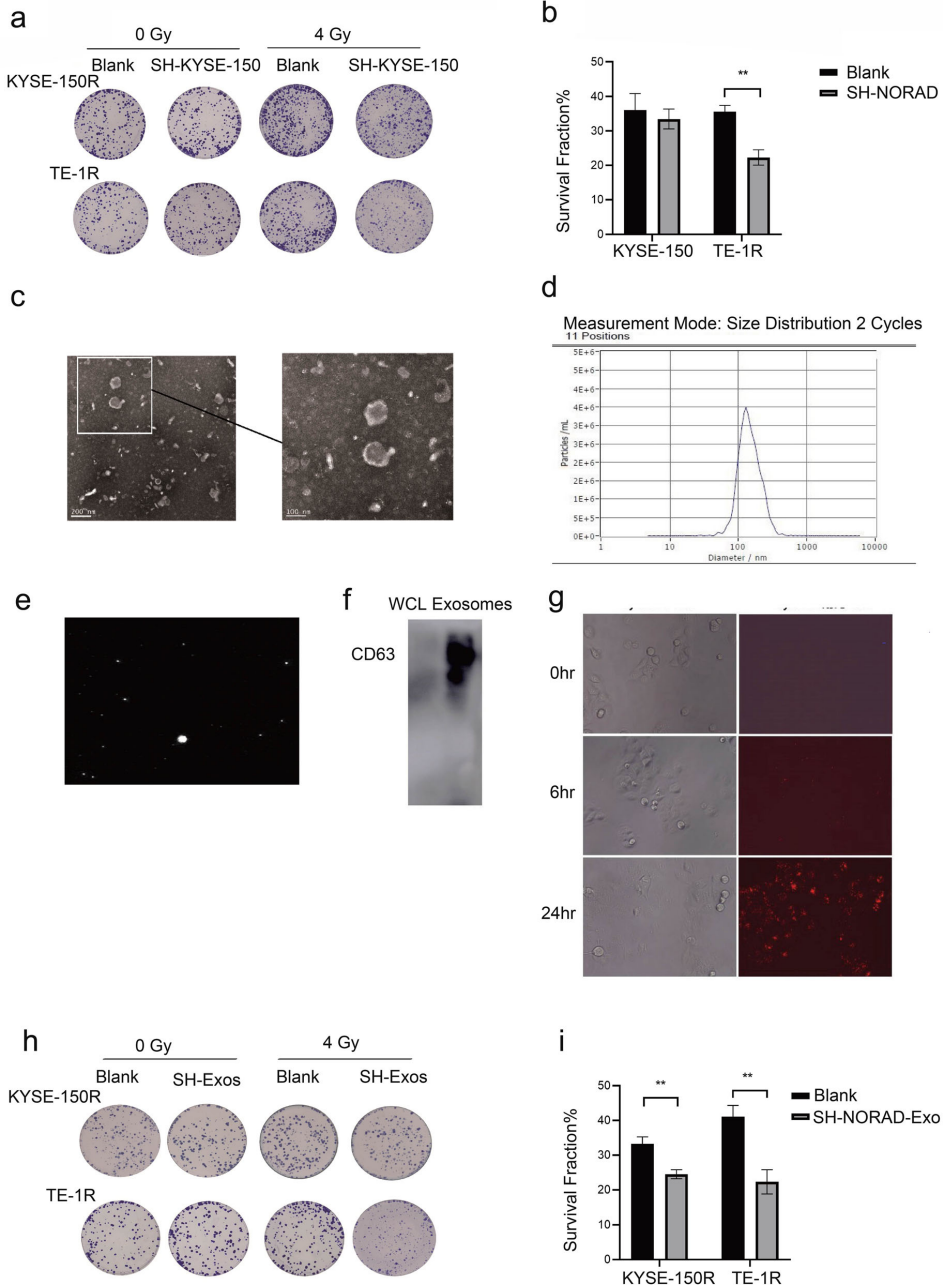
**a** KYSE-150R and TE-1R cells were cocultured with SH-KYSE-150 and SH-TE-1 cells respectively and then exposed to 0 and 4 Gy of X-ray radiation treatment. Colonies of KYSE-150R and TE-1R cells were stained and imaged after 14 days of radiation. **b** The survival fraction upon 4 Gy X-ray radiation of KYSE-150R and TE-1R cells that were cocultured with NORAD knockdown cells were calculated and analysed. **c** *Electron* microscopy was used to confirm the presence of exosomes extracted from SH-KYSE-150 cells. Scale bars represent 200 μm (left panel) and 100 μm (right panel). **d** NTA (nanoparticle tracking analysis) was used to assess the particle size and relative number of particles of exosomes extracted. **e** NTA (nanoparticle tracking analysis) exosome imaging with a representative picture of exosomes. **f** Western blot evaluating the level of the exosome marker CD63 in extracted exosomes. **g** Exosome tracer experiments to evaluate the ability of exosomes to enter target cells. **h** KYSE-150R and TE-1R cells were cocultured with SH-KYSE-150 exo and SH-TE-1 exo, respectively, and then exposed to 0 and 4 Gy of X-ray radiation treatment. Colonies of KYSE-150R and TE-1R cells were stained and imaged after 14 days of radiation. **i** The fractions of surviving KYSE-150R and TE-1R cells that were cocultured with NORAD knockdown exosomes and exposed to 4 Gy of X-ray radiation were calculated and analysed. (*n* = 3 technical replicates, *P < 0.05, **P < 0.01, *** *P* < 0.001, ****P < 0.0001)

Colony formation assays were adopted to verify the effect of NORAD on the radiosensitivity of KYSE-150 cells and TE-1 cells. A lower fraction of NORAD knockdown cells survived the 4 Gy X-ray treatment than control cells (Supplementary Fig. 3A-B). Furthermore, after coculture of NORAD knockdown cells with radiation-resistant cells (KYSE-150R and TE-1R), the radiosensitivity of radiation-resistant cells was increased (Fig. 5a-b). We speculated that NORAD knockdown cells might affect cocultured cells by releasing exosomes. We then extracted exosomes from NORAD knockdown cells, which were termed sh-NORAD exosomes. The extracellular vesicle-like structure of exosomes derived from NORAD knockdown cells was confirmed using electron microscopy (Fig. 5c); NTA (nanoparticle tracking analysis) was used to assess the particle size and relative number of particles, and CD63 expression confirmed their identity as exosomes (Fig. 5d-f). Exosome tracer experiments showed that sh-NORAD exosomes began to enter KYSE-150R cells after 6 h of incubation and were enriched in the cytoplasm after 24 h (Fig. 5g). Decreased fractions of surviving KYSE-150R and TE-1R cells were observed after treatment with sh-NORAD exosomes in response to 4 Gy radiation (Fig. 5h-i).

### NORAD knockdown sensitizes cocultured radioresistant ESCC cells to irradiation by promoting exosomal miR-199a-5p dispersion

**Fig. 6:**
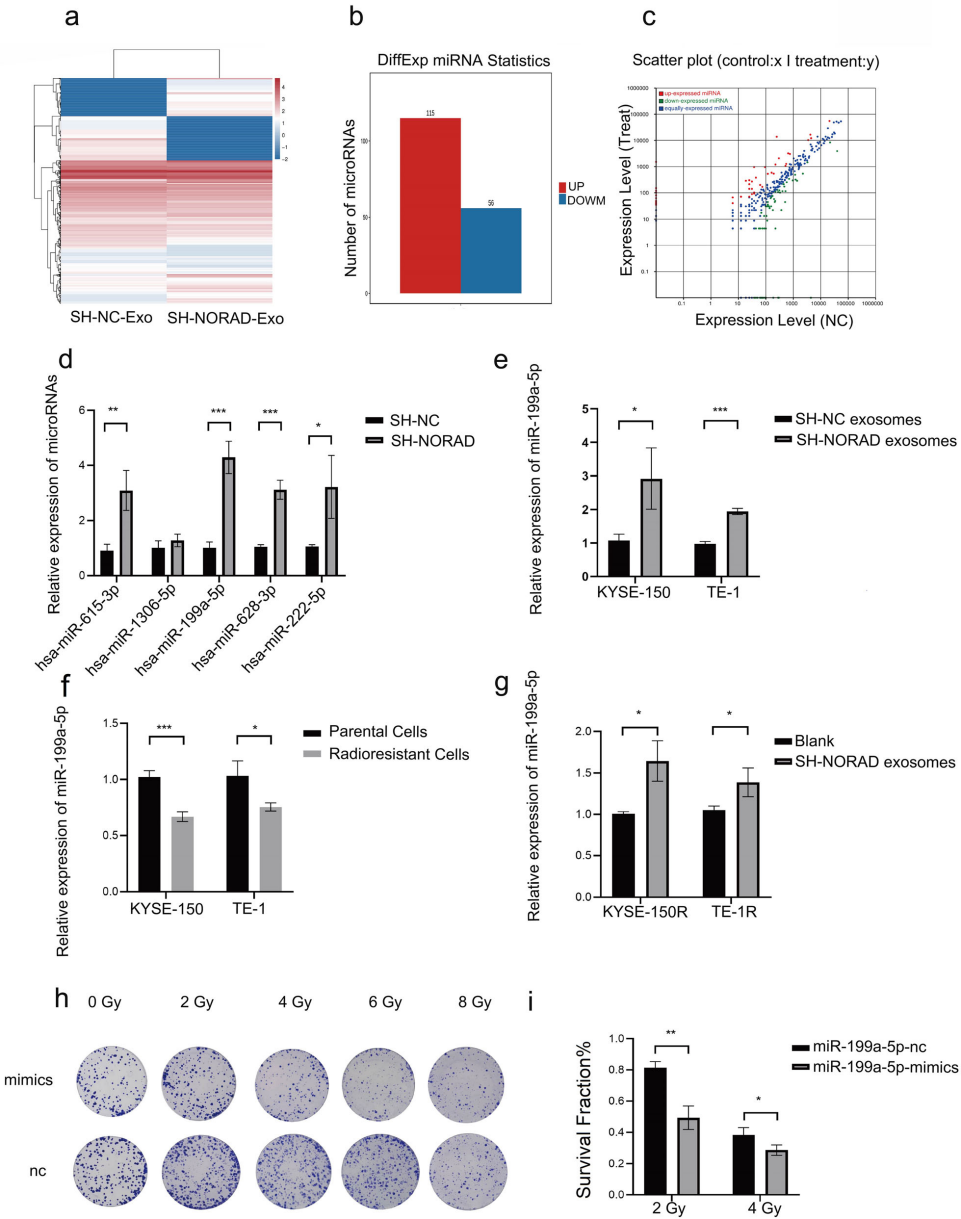
**a** Heatmap of the differentially expressed microRNAs between SH-NC exosomes and SH-NORAD exosomes. **b** The numbers of up- or downregulated microRNAs in SH-NORAD exosomes compared with SH-NC exosomes. **c** Scatter plot showing the expression of microRNAs in SH-NC exosomes and SH-NORAD exosomes. **d** qRT–PCR evaluation of the top 5 differentially expressed microRNAs (miR-615-3p, miR-1306-5p, miR-199a-5p, miR-628-3p, and miR-222-5p) in SH-NC-KYSE-150 cells and SH-NORAD-KYSE-150 cells. **e** qRT–PCR evaluation of the expression of miR-199a-5p in SH-NC exosomes and SH-NORAD exosomes. **f** qRT–PCR evaluation of the expression of miR-199a-5p in radioresistant ESCC cells (KYSE-150R and TE-1R) and their parental cells (KYSE-150 and TE-1). **g** qRT–PCR evaluation of the expression of miR-199a-5p in radioresistant ESCC cells (KYSE-150R and TE-1R) that were cocultured with or without SH-NORAD exosomes. **h** KYSE-150 cells were transduced with miR-199a-5p mimics or miR-199a-5p nc. Cells were exposed to 0, 2, 4, 6, 8 Gy of X-rays. Colonies were stained and images were captured after 14 days. **i** The survival fraction of KYSE-150R which were transduced with miR-199a-5p mimics or miR-199a-5p nc were calculated and analysed. (*n* = 3 technical replicates, *P < 0.05, **P < 0.01, *** *P* < 0.001, ****P < 0.0001. qRT–PCR results for miRNAs were quantified relative to U6)

Cancer-secreted exosomal miRNAs have been validated to regulate the crosstalk between cancer and stromal cells in numerous studies. Therefore, we investigated whether NORAD regulated cocultured cells via exosomal miRNAs. We next performed a microRNA-sequencing assay using exosomes from SH-NC-KYSE-150 and SH-NORAD-KYSE-150 cells. The differentially expressed microRNAs are presented in heatmaps (Fig. 6a). 115 miRNAs were upregulated and 56 miRNAs were downregulated in sh-NORAD exosomes (Fig. 6b). Differentially expressed miRNAs are presented in scatter plots (Fig. 6c). Significantly changed miRNAs were ranked by log_2_(FC) values and displayed (Supplementary Fig. 3C). We next examined the top 5 miRNAs in NORAD knockdown cells and control cells (Fig. 6d). qRT–PCR results confirmed that miR-199a-5p was significantly overexpressed in sh-NORAD exosomes (Fig. 6e). KYSE-150R and TE-1R cells were cultured with sh-NORAD exosomes to investigate whether exosomal miR-199a-5p was delivered to co-cultured cells. qRT–PCR results showed that miR-199a-5p was downregulated in KY-SE150R and TE-1R cells compared with parental control cells. Coincubation resulted in increased expression of miR-199a-5p in KY-SE150R and TE-1R cells (Fig. 6f-g). Colony formation assay results confirmed that overexpression of miR-199a-5p sensitized KYSE-150R cells to radiation (Fig. 6h-i). Based on these results, NORAD knockdown cells resensitize radioresistant cells to radiation via the exosomal miR-199a-5p pathway.

### NORAD inhibits pri-miR-199a1 processing by competitively binding PUM1

**Fig. 7:**
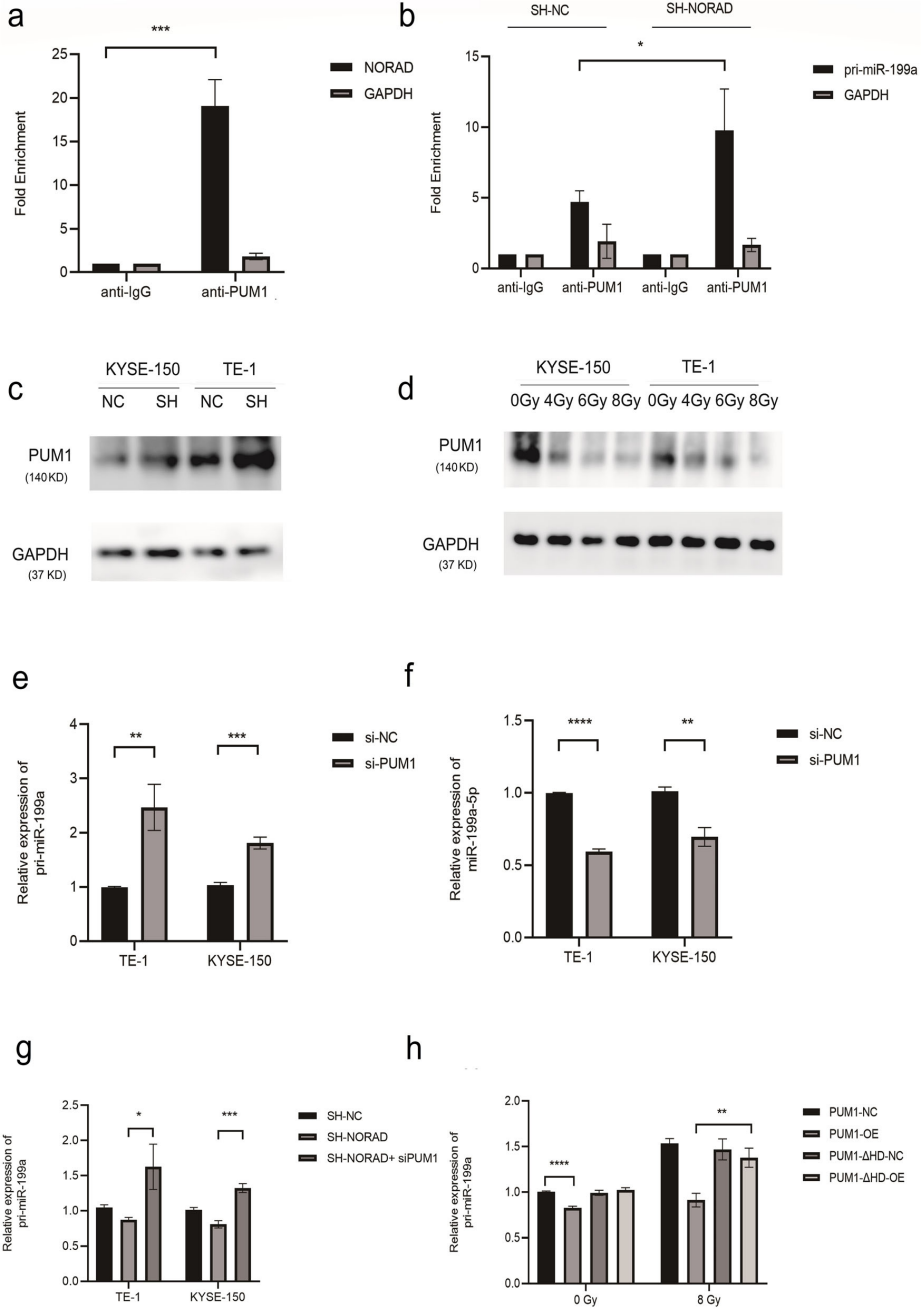
**a-b** RIP -qPCR assay of KYSE-150 cells was performed using an anti-PUM1 or control IgG antibody to detect NORAD expression (**a**) and pri-miR-199a1 expression (**b**). Values were normalized to the levels immunoprecipitated with the normal control IgG. **c** PUM1 protein expression was measured by using western blot in KYSE-150 and TE-1 cells transfected with SH-NORAD or SH-NC. **d** Effects of 0, 4 Gy, 6 Gy, and 8 Gy of X-ray radiation treatment on PUM1 protein expression were measured by using western blotting in KYSE-150 and TE-1 cells. **e** qRT–PCR assay to evaluate the expression of pri-miR-199a1 in KYSE-150 cells transduced with si-PUM1 or si-NC. **f** qRT–PCR assay to evaluate the expression of miR-199a-5p in KYSE-150 cells transduced with si-PUM1 or si-NC. **g** qRT–PCR assay to evaluate the expression of pri-miR-199a1 in NORAD knockdown cells and NORAD knockdown cells transduced with si-PUM1. **h** The effect of PUM1 or PUM1-ΔHD overexpression on pri-miR-199a1 expression in KYSE-150 cells treated with or without 8 Gy of X-ray radiation. (*n* = 3 technical replicates, *P < 0.05, **P < 0.01, *** *P* < 0.001, ****P < 0.0001, qRT–PCR results for miRNAs were quantified relative to GAPDH)

No complementary sequence existed between miR-199a-5p and NORAD. Based on ENCODE datasets, the ChIP-Seq analysis using the anti-PUM1 antibody in K562 cells revealed that PUM1, which was regulated by NORAD, bound pri-miR-199a1 (Supplementary Fig. 3D). RIP-qPCR results confirmed the interaction of PUM1 with NORAD (Fig. 7a) and pri-miR-199a1 (Fig. 7b). NORAD knockdown enhanced the interaction between PUM1 and pri-miR-199a1 (Fig. 7b). We speculated that NORAD regulated miR-199a-5p by modulating PUM1. Western blot analysis showed that NORAD knockdown increased PUM1 expression (Fig. 7c). Radiation treatment decreased PUM1 expression (Fig. 7d). PUM1 knockdown significantly increased pri-miR-199a1 expression but decreased mature miR-199a-5p expression (Fig. 7e-f). qRT–PCR results showed that the decreased expression of pri-miR-199a1 induced by NORAD knockdown was rescued by PUM1 knockdown (Fig. 7g). Because PUM1 bound and processed RNA through its HD region, we next constructed plasmids to overexpress PUM1 or a PUM1 mutant lacking the RNA-binding domain (termed PUM1-ΔHD). The expression of pri-miR-199a1 was decreased in cells overexpressing PUM1 but not PUM1-ΔHD. Treatment with 8 Gy X-ray radiation upregulated the expression of pri-miR-199a1; overexpression of PUM1 but not PUM1-ΔHD inhibited the upregulation of pri-miR-199a1 by ionizing radiation (Fig. 7h). Together, these results confirmed that NORAD delays the processing of pri-miR-199a by competitively binding PUM1.

### NORAD knockdown impairs homologous recombination repair by downregulating EEPD1

**Fig. 8:**
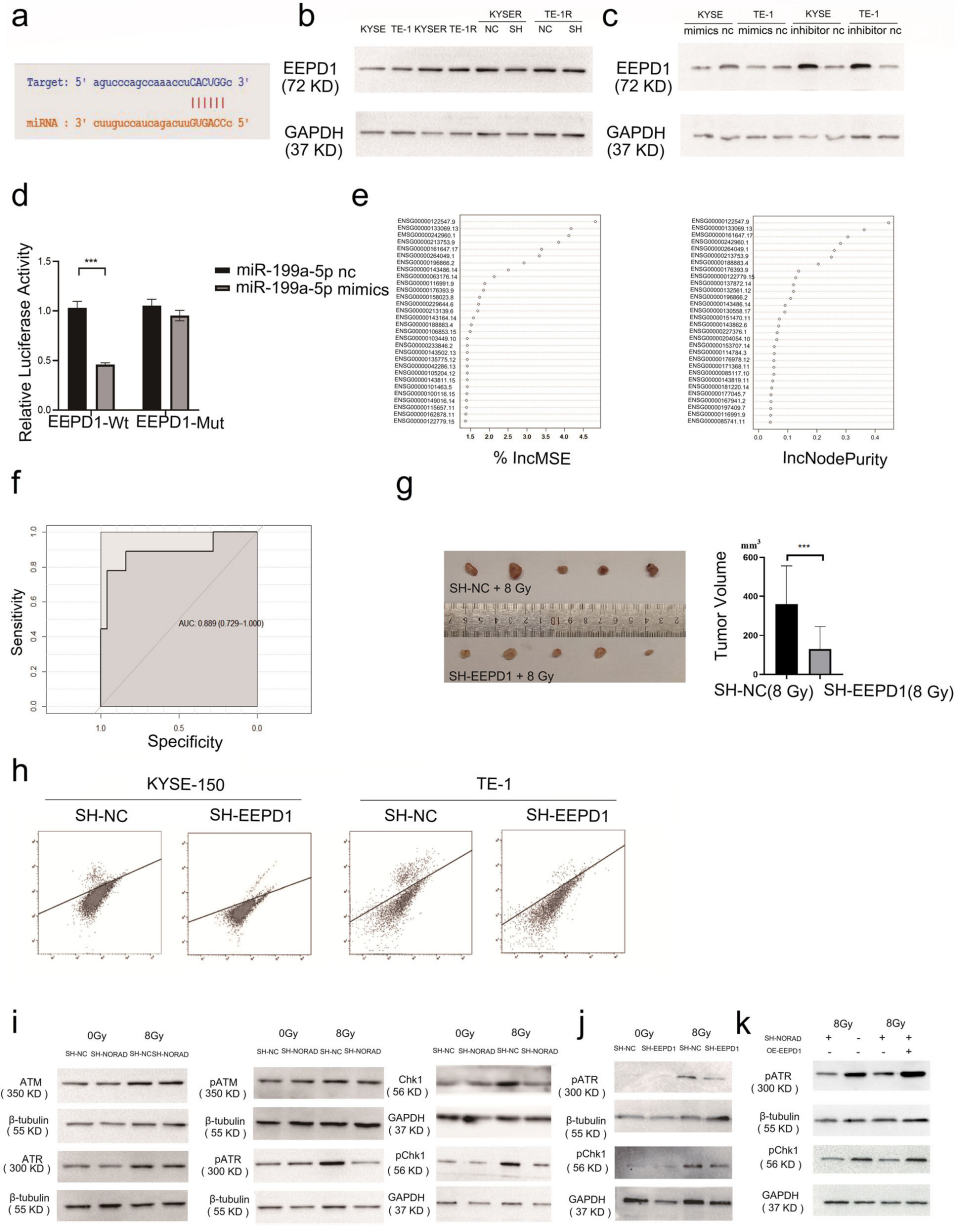
**a** The starBase database predicted the complementary sequences between miR-199a-5p and NORAD. **b** EEPD1 expression in radioresistant ESCC cells (KYSE-150R and TE-1R) and their parental cells (KYSE-150 and TE-1) was measured using western blotting. The effects of NORAD knockdown on EEPD1 expression in KYSE-150R and TE-1R cells were measured using western blotting. **c** EEPD1 expression in KYSE-150 and TE-1 cells transduced with miR-199a-5p mimics or miR-199a-5p inhibitors, respectively, was measured using western blotting. **d** Dual-luciferase reporter assay conducted by cotransfecting the wild-type or mutant 3’UTR EEPD1 mRNA with the miR-199a mimic or negative control into KYSE-150 cells. **e** Relative importance of genes for segregating patients with radioresistant ESCC from radiosensitive patients calculated using the random forest algorithm. The importance of each gene was ranked by its IncMSC values and IncNodePurity values. **f** ROC curve for segregating patients with radioresistant ESCC from radiosensitive patients based on EEPD1 expression. **g** The effect of EEPD1 on radiotherapy outcomes in ESCC xenografts. Subcutaneous ESCC xenografts were established with SH-EEPD1 cells and SH-NC cells. Tumours were exposed to 8 Gy of X-ray radiation. The tumours were harvested and imaged at 3 weeks after radiotherapy delivery; tumour volumes were calculated and analysed. **h** KYSE-150 and TE-1 cells were transduced with an I-SceI expression plasmid to induce DSBs, and homologous recombination repair in EEPD1 knockdown cells and control cells was assayed using flow cytometry and is presented as the percentage of GFP-positive cells. **i** Levels of ATM, ATR, Chk1 and phosphorylated ATM, ATR, and Chk1 were analysed in NORAD knockdown cells and control cells treated with or without 8 Gy of X-ray using western blotting. **j** Levels of phosphorylation of ATR and Chk1 were analysed in EEPD1 knockdown cells and control cells treated with or without 8 Gy of X-rays using western blotting. **k** Levels of phosphorylation of ATR and Chk1 were analysed using western blotting in KYSE-150-SH-NORAD cells with or without EEPD1 overexpression after 8 Gy of MV X-ray treatment

We performed TMT mass spectrometry to quantify the protein levels in SH-NORAD-KYSE-150 and SH-NC-KYSE-150 and to obtain further insights into the potential proteins regulated by NORAD (Supplementary Fig. 4A). The top 30 differentially expressed proteins are presented in a heatmap (Supplementary Fig. 4B). GO and KEGG analyses of differentially expressed proteins were performed (Supplementary Fig. 4C-D). Simultaneously, a starBase dataset was adopted to predict the potential targets of miR-199a-5p. Among the potential targets of miR-199a-5p, we found that EEPD1, the gatekeeper for the repair of stressed replication forks, was downregulated in NORAD knockdown cells based on the TMT mass spectrometry results (Fig. 8a). Western blot results showed that EEPD1 was upregulated in radioresistant ESCC cells and that NORAD knockdown decreased EEPD1 expression (Fig. 8b and Supplementary Fig. 1D). STRING and KEGG analyses of EEPD1 indicated that EEPD1 had crucial functions in the DNA damage repair process (Supplementary Fig. 4E-F). IHC staining of xenograft tumours showed that tumours derived from NORAD knockdown cells exhibited less EEPD1 staining (Supplementary Fig. 5A). Furthermore, miR-199a-5p mimic transduction decreased the expression of EEPD1, and miR-199a-5p inhibitors upregulated EEPD1 in ESCC cells (Fig. 8c). Transfection of the miR-199a-5p mimics significantly decreased the relative luciferase activity of EEPD1-Wt but failed to influence the relative luciferase activity of EEPD1-Mut, indicating that miR-199a-5p interacts with the 3′UTR of the EEPD1 mRNA (Fig. 8d).

Thirty-four patients with ESCC and a radiotherapy response evaluation in TCGA datasets were next analysed. Based on the radiotherapy response, 25 patients were placed in the radiosensitive group (complete response), and 9 patients were placed in the radioresistant group (partial response, stable disease or radiographic progressive disease). The clinical characteristics of the patients in the two groups are shown in Supplementary Table 1. Because different TNM stages and tumour locations might influence the ESCC radiotherapy outcome, we used the chi-square test to compare these clinical characteristics between the two groups, and no significant differences were found (Supplementary Table 1). The importance of each gene in ESCC radioresistance was ranked by the IncMSC and IncNodePurity values, which were calculated using the random forest algorithm. Notably, the IncMSC and IncNodePurity values of EEPD1 were obviously higher than those of the other genes (Fig. 8e). Based on EEPD1 expression, the area under the ROC curve (AUC) for segregating radioresistant patients from radiosensitive patients was 0.889 (95% CI 0.729–1.000) (Fig. 8f).

We then examined EEPD1 expression by performing IHC of samples from 77 patients who received adjuvant chemo-radiotherapy after esophagectomy. The clinical characteristics of the patients are shown in Supplementary Table 2. Patients with local recurrence after radiotherapy were included in the chemo-radiotherapy group. No significant differences in clinical characteristics were observed between the two groups (Supplementary Table 2). IHC results showed that EEPD1 was mainly located in the nucleus (Supplementary Fig. 5B). Among patients with chemo-radiotherapy resistant ESCC, 20 (20/29) showed high EEPD1 expression. The results of the χ^2^ test showed that high EEPD1 expression was associated with radiation resistance (χ^2^ = 8.151; *p* = 0.004**; Supplementary Table 3). Xenograft tumours from EEPD1 knockdown and control cells showed that EEPD1 knockdown significantly sensitized ESCC cells to radiation in vivo (Fig. 8g). The HR reporter assay showed that EEPD1 knockdown cells presented lower HR rates after DNA double-strand breaks (Fig. 8h). EEPD1 knockdown inhibited ATR and Chk1 phosphorylation upon radiation treatment, consistent with the NORAD knockdown cell phenotype (Fig. 8i-j). EEPD1 overexpression restored the phosphorylation of ATR-Chk1, which was reduced in NORAD knockdown cells after 8 Gy MV X-ray treatment (Fig. 8k).

### High NORAD expression predicts radioimmunotherapy failure in vivo

**Fig. 9:**
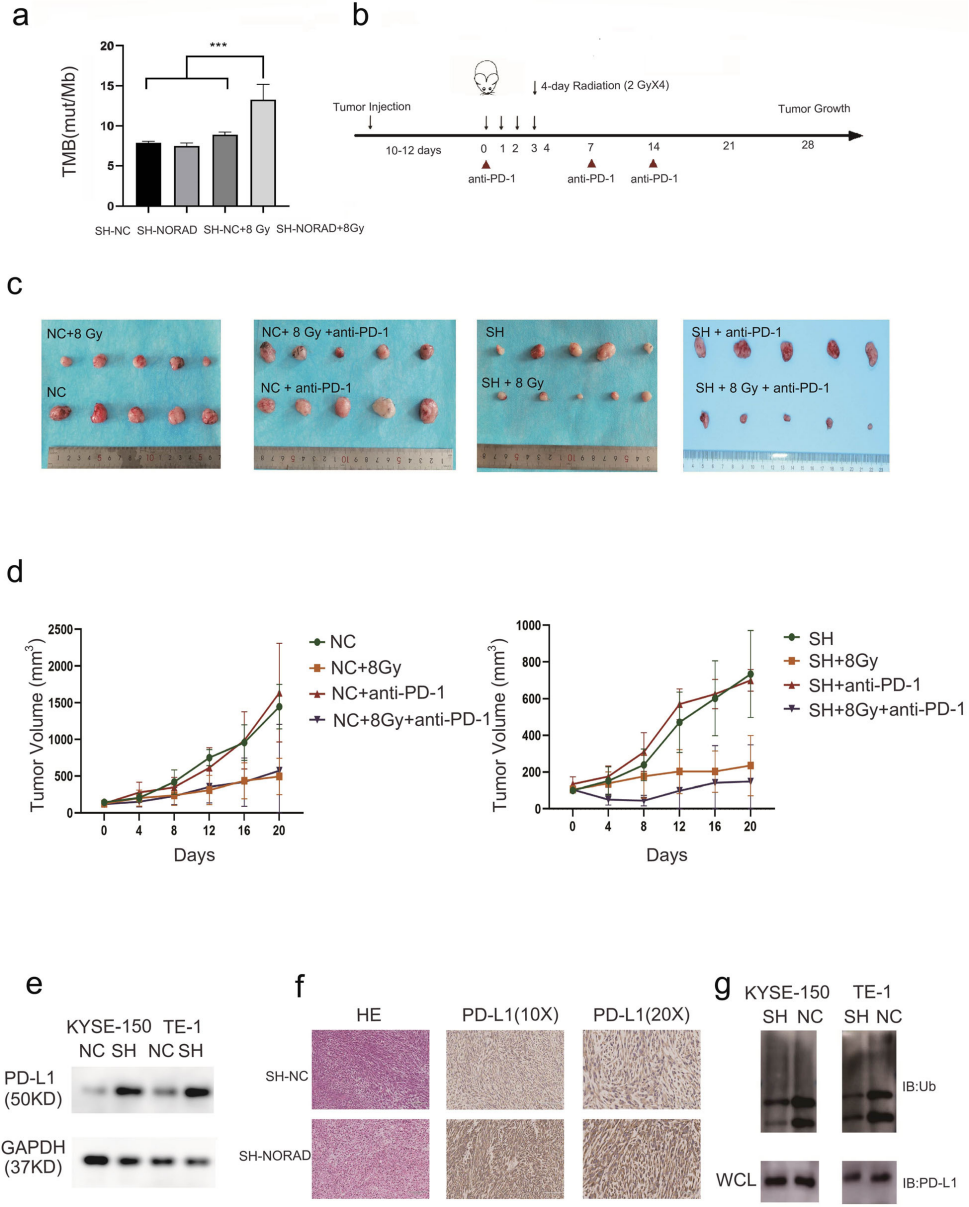
**a** SH-NC-AKR and SH-NORAD-AKR cell-derived xenograft tumours were exposed to 0 or 8 Gy of X-ray radiation. The tumour mutation burden was analysed using whole exome sequencing. **b** Scheme for treatments and analyses. Radiation (8 Gy) was delivered in 4 fractions on consecutive days. The anti-PD-1 antibody was administered on the day of the first radiation treatment and thereafter once per week. **c** Subcutaneous ESCC xenograft tumours derived from AKR-SH-NC cells or AKR-SH-NORAD cells. C57BL/6 mice were treated with anti-PD-1, 4 × 2 Gy of radiation or combination therapy. **d** The growth curves of tumours shown in **c**. **e** PD-L1 expression was analysed using western blotting in SH-NC cells and SH-NORAD cells. **f** Representative images of IHC staining for PD-L1 in ESCC xenograft tumours derived from AKR-SH-NC cells or AKR-SH-NORAD cells. Scale bars in the images of IHC staining for PD-L1 (10×) represented 200 μm and in the images of IHC staining for PD-L1 (20×) represented 100 μm. **g** Western blot measurement of PD-L1 pull-down of ubiquitin derived from the lysates of NORAD knockdown and control cells. Cells were pretreated with MG132 (20 μM) for 12 h

Considering that radiation led to severe DNA damage in cells and NORAD knockdown increased genomic instability upon DNA damage, we reasoned that NORAD knockdown increased the tumour mutation burden (TMB) in ESCC upon radiation. In a xenograft tumour model, radiation or NORAD knockdown alone did not change the TMB. However, NORAD knockdown combined with radiotherapy increased the TMB (Fig. 9a). According to previous studies, the tumour mutation burden (TMB) may predict the clinical response to immune checkpoint inhibitors. We investigated whether NORAD knockdown combined with radiation enhanced anti-PD-1 efficiency in C57BL/6 mice. The combination treatment consisted of anti-PD-1 therapy and 4 fractions of 8 Gy radiation on consecutive days (Fig. 9b). NORAD knockdown significantly sensitized tumours to radiation, but anti-PD-1 monotherapy was not sufficient to control tumour growth. Compared with radiation treatment alone, radiation combined with anti-PD-1 therapy suppressed tumour growth in the NORAD knockdown group but not in the control group (Fig. 9c-d). Thus, NORAD might affect the efficiency of radiotherapy combined with anti-PD-1 treatment. We next examined PD-L1 expression in NORAD knockdown cells. NORAD knockdown cells displayed elevated PD-L1 protein expression (Fig. 9e), and IHC staining of xenograft tumours showed that tumours derived from NORAD knockdown cells exhibited stronger PD-L1 staining (Fig. 9f) than tumours derived from control cells. We treated cells with the proteasome inhibitor MG132, and subsequent Co-IP experiments indicated that the levels of PD-L1 ubiquitination were decreased in NORAD knockdown cells (Fig. 9g). Based on these results, NORAD knockdown inhibits the ubiquitination of PD-L1. By subsequently evaluating the tumour-infiltrating lymphocytes, however, we did not observe a significant change in tumour-infiltrating lymphocytes between the NORAD knockdown mouse group and the control group (Supplementary Fig. 6A). Using CIBERSORT, we found that NORAD expression did not affect immune cell infiltration (Supplementary Fig. 6B). NORAD influenced the immunogenic function of radiation but not the tumour immune microenvironment. Taken together, these results suggest that NORAD is a potential treatment target for improving the efficacy of immunotherapy in patients with ESCC.

## Discussion

Previously, our group identified that lncRNA-NORAD, whose expression is induced by DNA damage, plays key roles in mediating radiation resistance. NORAD is overexpressed in ESCC both in vivo and in vitro; furthermore, NORAD knockdown sensitizes ESCC cells to radiation treatment. In this manuscript, we further investigated the corresponding molecular mechanisms. NORAD delays pri-miR-199a1 maturation by inhibiting the interaction of PUM1 with pri-miR-199a1 and subsequently releasing EEPD1, which is recruited to damaged DNA and promotes HR in response to radiation. Concurrently, NORAD knockdown inhibits PD-L1 ubiquitination and enhances anti-PD-1 treatment efficacy, especially when administered in combination with radiotherapy. Our results indicate that NORAD represents a novel target for improving the efficacy of radiation and anti-PD1 therapy by regulating the DNA damage response.

The HR reporter assay results confirm that NORAD knockdown sensitizes ESCC to radiation by reducing HR efficiency. We subsequently confirmed that NORAD knockdown delays the HR process by inhibiting ATR/Chk1 signalling activation in response to radiation. The inhibition of DNA repair components, including ATM, ATR, Chk1 and Chk2, markedly sensitizes cancer cells to radiation [23–25]. For example, Teng et al. found that inhibiting either ATM or ATR significantly enhances the radiation response of gynaecological cancer cells (ovarian, endometrial, and cervical cancer cells) [26]. Notably, the generation of double-strand breaks is the central mechanism of action of radiotherapy. In response to radiation-induced DNA damage, ATM, ATR and DNA-PK_S_, three important DNA damage sensors, are immediately activated and target a variety of overlapping substrates that promote DNA repair, cell cycle arrest and apoptosis [27, 28]; these biological processes help cancer cells escape radiation damage. Under radiation stress, NORAD knockdown significantly increased ESCC cell apoptosis rates and impaired arrest at G2 phase, leading to genomic instabilities in ESCC cells. Based on these results, we inferred that NORAD is an effective target for enhancing the cytotoxic effects of radiation on ESCC.

Two major mechanisms have been reported for NORAD-mediated regulation of genomic stability. A recent study revealed that NORAD functions as an RNA-binding molecule; interacts with RBMX, TOP1 and other proteins; and facilitates the formation of the topoisomerase complex [11]. An earlier study confirmed that NORAD maintained genomic stability by binding to and negatively regulating PUMILIO (PUM1 and PUM2) in the cytoplasm [10, 29]. In the present study, NORAD was mainly located in the cytoplasm of ESCC cells. FISH results confirmed that cytoplasmic NORAD expression but not nuclear NORAD expression was significantly increased in response to radiation. Previous studies have indicated that nuclear lncRNAs tend to regulate transcription in cis or trans, organize subnuclear structures, and mediate chromosomal interactions [30, 31]; cytoplasmic lncRNAs are known to modulate the activity or abundance of interacting proteins or mRNAs [9, 32, 33]. Studies on the sublocalization of NORAD in cells have not produced consistent results. Lee et al. reported that NORAD mainly exists in the cytoplasm. Cytoplasmic NORAD negatively regulates the PUM protein and maintains the stability of the cell genome by binding the PUM protein. Other studies have reported that adriamycin causes the nuclear translocation of NORAD, and the results of proteomic analysis of the NORAD interaction group showed that RBMX is the main protein interacting with NORAD. Subsequent experiments showed that the NORAD/RBMX interaction promotes the assembly of ribonucleoprotein (RNP) complexes in the nucleus, including topoisomerase I (TOP1) and other proteins crucial to genome maintenance, to maintain the stability of the cell genome [11]. The mechanisms in the two studies were different, the binding sites of PUM and RBMX on NORAD were different, and they might play roles in the cytoplasm and nucleus. Therefore, although the localization of NORAD in cells remains to be clarified, both PUM and RBMX may play important and not mutually exclusive roles in the function of NORAD in maintaining genome stability. NORAD knockdown significantly increases the sensitivity of cells to DNA-damaging drugs and leads to chromosomal instability (CIN).

As an RNA-binding protein, PUM1 binds to RNAs containing the PUMILIO response element (UGUANAUA or UGUANAUN), thus mediating RNA deadenylation, decapping, and degradation [29]. We found that PUM1 bound to pri-miR-199a1 and facilitated processing of pri-miR-199a1. Notably, miRNAs are transcribed initially as pri-miRNAs and recognized by RNase III DROSHA and RNA-binding protein DGCR8 in the nucleus. The flanking single-stranded sequences are cleaved by DROSHA and generate pre-miRNAs, which are then cleaved by the RNase III Dicer to generate double-stranded RNA of ~ 21 nucleotides (nt) in length. Recently, numerous studies have reported that the expression level of mature miRNA is driven by both transcription and processing rates during the Drosha and Dicer processes [34]. For example, Guil et al. stated that hnRNP A1, an RNA-binding protein, specifically bound to pri-miR-18a before Drosha processing [35]. Another stem cell factor, LIN28, interacts with pre-miRNAs of the let-7 family and recruits uridyltransferases to these pre-miRNAs, essentially blocking their expression [36]. Here, we found that PUM1 binds to pri-miR-199a1 and functions as an auxiliary factor for the Drosha processing of pri-miR-199a1. This interaction is inhibited by NORAD, as upregulation of NORAD significantly delays Drosha processing of pri-miR-199a1 by sequestering PUM1 in the cytoplasm. Notably, NORAD knockdown not only facilitates the processing of miR-199a-5p by inhibiting PUM1 but also restores the radiosensitivity of cocultured radioresistant ESCC cells. NORAD knockdown drives the transfer of exosomal miR-199a-5p from NORAD knockdown cells to radioresistant cells. In addition, EEPD1 is downregulated in radioresistant ESCC cells. Taken together, these results expanded the potential clinical importance of NORAD in ESCC radiotherapy.

With the assistance of random forest algorithm, we identified EEPD1 as a powerful biomarker for predicting the radioresistance of ESCC. Wu et al. previously reported that EEPD1 is recruited to stalled forks in response to DNA damage, where it promotes the restarting of these stalled forks by resecting the 5′ DNA end near the fork junction, thus permitting the invasion of the 3′ single strand and the initiation of HR. [37] Hyun-Suk Kim et al. reported that EEPD1 functions as a gatekeeper for HR; it cleaves replication forks and creates a binding site for Exo1 on the free 5′ DNA end [20]. Furthermore, Changzoon Chun et al. described similar biological functions for EEPD1 and BRCA1. Depletion of EEPD1 in stressed zebrafish embryos results in chromosomal abnormalities, including anaphase bridges and micronuclei [38]. These results confirm that EEPD1 functions in the initiation of HR and that deleting EEPD1 sensitizes cells to DNA replication stressors.

Interestingly, NORAD knockdown also enhances the efficacy of immune checkpoint inhibitors in combination with radiotherapy in tumour treatment. NORAD knockdown upregulates PD-L1 expression by inhibiting its ubiquitination. These phenomena may suggest the poor response of patients with radioresistant ESCC to immune checkpoint inhibitors. According to one study, PD-L1 protein expression fluctuates during the cell cycle and peaks at G1 phase. Researchers have reported that PD-L1 is regulated by the G1 cycle checkpoint Cyclin D-CDK4 via the Cullin3^SPOP^-E3 ligase pathway [39]. Here, NORAD knockdown induced ESCC cell arrest at G1 phase in the cell cycle, which might be responsible for the observed PD-L1 upregulation. Only a limited number of patients with ESCC benefit from anti-PD1 therapy. In the most recent prospective clinical trial, pembrolizumab did not improve overall survival compared with paclitaxel as a second-line therapy for advanced esophageal cancer [40]. Radiation administered both alone and in combination with anti-PD-1 treatment suppressed tumour growth, but the combination did not significantly improve the tumour response compared to that of radiation monotherapy. For mice bearing AKR-sh-NORAD tumours, the use of combination therapy significantly inhibited tumour growth compared radiation monotherapy. Notably, NORAD knockdown facilitates the antitumour efficiency of anti-PD-1 treatment and radiation in ESCC. These results reveal that NORAD is a potential treatment target for improving immunotherapy efficacy in patients with ESCC. Conceptually, inhibiting DNA damage repair increases the efficacy of radiotherapy in combination with immune checkpoint inhibitors in patients with ESCC.

## Conclusions

Radioresistance frequently leads to local recurrence and treatment failure in patients with esophageal squamous cell carcinoma. Here, we identified lncRNA-NORAD is crucial for mediating esophageal squamous cell carcinoma radioresistance. NORAD is epigenetically activated by irradiation via enhanced H3K4me2 enrichment. High cytoplasmic NORAD levels sequester PUM1, preventing the interaction of PUM1 with pri-miR-199a1 and delaying pri-miR-199a1 processing and miR-199a-5p maturation. NORAD knockdown cells drive miR-199a-5p assembly into exosomes and sensitize cocultured radioresistant cells by targeting the key protein in homologous recombination EEPD1 and the ATR/Chk1 signalling pathway. Remarkably, by increasing genomic instability through DNA damage, NORAD knockdown resulted in a better response of mice to radiation therapy in combination with anti-PD-1. Our work suggests that NORAD is an effective treatment target that might enhance the cytotoxic effects of radiation and potentiate the immunogenic function of radiation in patients with ESCC.

## Supplementary Information

**Additional file 1: Supplementary Fig. 1. A.** RNA FISH results for NORAD in ESCC tissues. Scale bars represent 5000 μm (left panel) and 50 μm (right panel). **B.** The enrichment of H3K4me2, H3K4me3, and H3K9ac in the NORAD region in HCT116, HeLa-S3, HepG2, MCF-7, SK-N-SH and K562 cells and the enrichment of H3K14ac and H3K18ac in the NORAD region in neuronal stem cells and mesenchymal stem cells. Analyses were performed using the UCSC database. **C.** Densitometry analysis of H3K4me2 expression in figure3B. **D.** Densitometry analysis of EEPD1 expression in Figure8B.

**Additional file 2: Supplementary Fig. 2.** The Pearson correlation coefficients for NORAD expression with H3K4 methyltransferases (KMT2A, KMT2B, KMT2C, KMT2D, Set1A, Set1B, ASH2L, RBbp5, WDR5 and DPY30). Analyses were performed using GEPIA (http://gepia.cancer-pku.cn/).

**Additional file 3: Supplementary Fig. 3. A.** KYSE-150 and TE-1 cells were transfected with SH-NORAD or SH-NC and exposed to 0 and 4 Gy of X-ray radiation. Colonies of cells were stained and imaged after 14 days of radiation. **B.** The fraction of surviving cells in Sup. Figure 3A upon 4 Gy X-ray radiation was calculated and analysed. **C.** Top 17 differentially expressed microRNAs between SH-NC exosomes and SH-NORAD exosomes. Columns represent the log2(fc) of two biological experiments. **D.** ChIP-seq peaks of PUM1 at the pri-miR-199a1 region in K562 cells, as displayed using the UCSC Genome Browser.

**Additional file 4: Supplementary Fig. 4. A.** SDS–PAGE analysis of total proteins extracted from KYSE-150-sh-NC and KYSE-150-sh-NORAD cells. **B.** Heatmap of differentially expressed proteins between KYSE-150-sh-NC and KYSE-150-sh-NORAD cells. **C.** KEGG analysis of the differentially expressed proteins in NORAD knockdown cells. **D.** Gene Ontology analysis of the differentially expressed proteins in NORAD knockdown cells. **E.** The protein–protein interactions for the potential interacting proteins of EEPD1 were mapped using the STRING (Search Tool for the Retrieval of Interacting Genes/Proteins) database (version 11.0). **F.** Gene Ontology analysis of proteins potentially interacting with EEPD1.

**Additional file 5: Supplementary Fig. 5. A.** Representative IHC for EEPD1 in xenografts tumors derived from NORAD knockdown and control cells. Scale bar represented 100μm (Left) and 200μm (Right) respectively. **B.** Immunohistochemistry staining of EEPD1 in 77 ESCC cancer tissues. Scale bar represented 2000 μm (Upper) and 50μm (Lower) respectively.

**Additional file 6: Supplementary Fig. 6. A.** CIBERSORT results of the fraction of infiltrating immune cells (B cells, CD4+ T cells, CD8+ T cells and macrophages) in groups with high NORAD expression and low NORAD expression. The two groups were separated according to the mean value of NORAD expression. Data were extracted from TCGA datasets. **B.** Representative images of IHC staining for CD8+ T cells and CD4+ T cells in SH-NC-AKR and SH-NORAD-AKR cell-derived tumours. The scale bar in IHC images (10×) represents 200 μm, and the scale bar in other images (20×) represents 100 μm.

**Additional file 7: Table S1.** Clinical characteristics of ESCC patients in radio-resistant group and the radio-sensitive group in TCGA datasets.

**Additional file 8: Table S2.** Clinical characteristics of 77 ESCC patients in chemoradiotherapy resistant group and the chemoradiotherapy sensitive group.

**Additional file 9: Table S3.** The correlation between EEPD1 expression and radiotherapy outcome in 77 ESCC patients.

## Data Availability

Data and materials are available upon reasonable request if applicable.

## Abbreviations

EAC: Esophageal Adenocarcinoma
ESCC: Esophageal Squamous Cell Carcinoma
TMT Mass Spectrometry: Tandem Mass Tag Mass Spectrometry
HR: Homologous Recombination
NEJM: Non-homologous End Joining (NHEJ)
DR-GFP: Reporter Direct Repeat - Green Fluorescent Protein Reporter
NTA: Nanoparticle Tracking Analysis
WGCNA: Weighted Gene Coexpression Network Analysis
KEGG: Kyoto Encyclopedia of Genes and Genomes
GO: Gene Ontology
STRING: Search Tool for the Retrieval of Interacting Genes/Proteins
IncMSC: Increase in MSE
IncNodePurity: Increase in NodePurity
