# Supplementary figures and images for: Radiation induces NORAD expression to promote ESCC radiotherapy resistance via EEPD1/ATR/Chk1 signalling and by inhibiting pri-miR-199a1 processing and the exosomal transfer of miR-199a-5p
Source: J Exp Clin Cancer Res. 2021 Sep 29;40:306. doi: 10.1186/s13046-021-02084-5 (PMC8479908; doi:10.1186/s13046-021-02084-5)

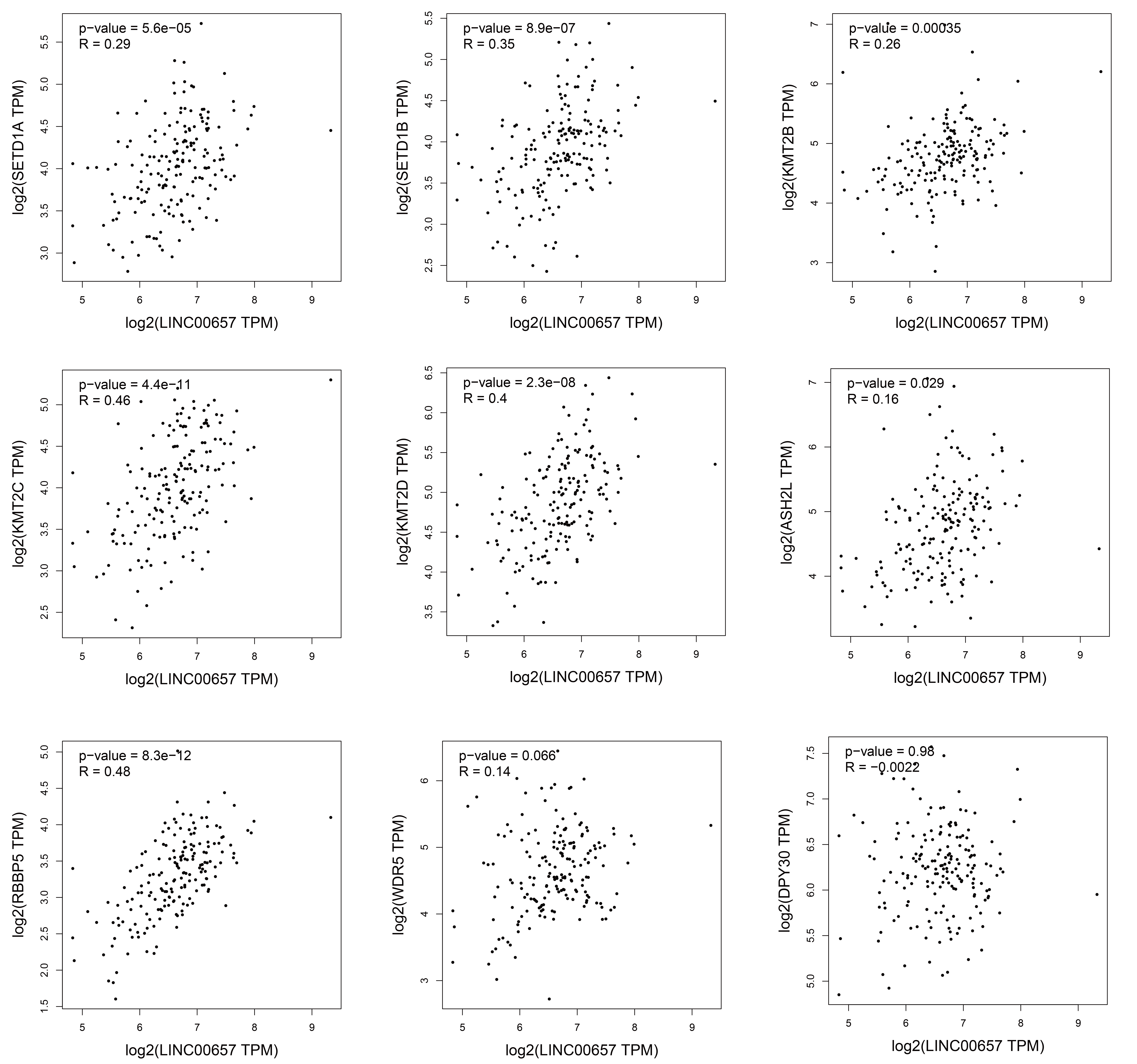

Supplement: Supplementary file 2 — Additional file 2: Supplementary Fig. 2. The Pearson correlation coefficients for NORAD expression with H3K4 methyltransferases (KMT2A, KMT2B, KMT2C, KMT2D, Set1A, Set1B, ASH2L, RBbp5, WDR5 and DPY30). Analyses were performed using GEPIA (http://gepia.cancer-pku.cn/). [file 13046_2021_2084_MOESM2_ESM.tiff]

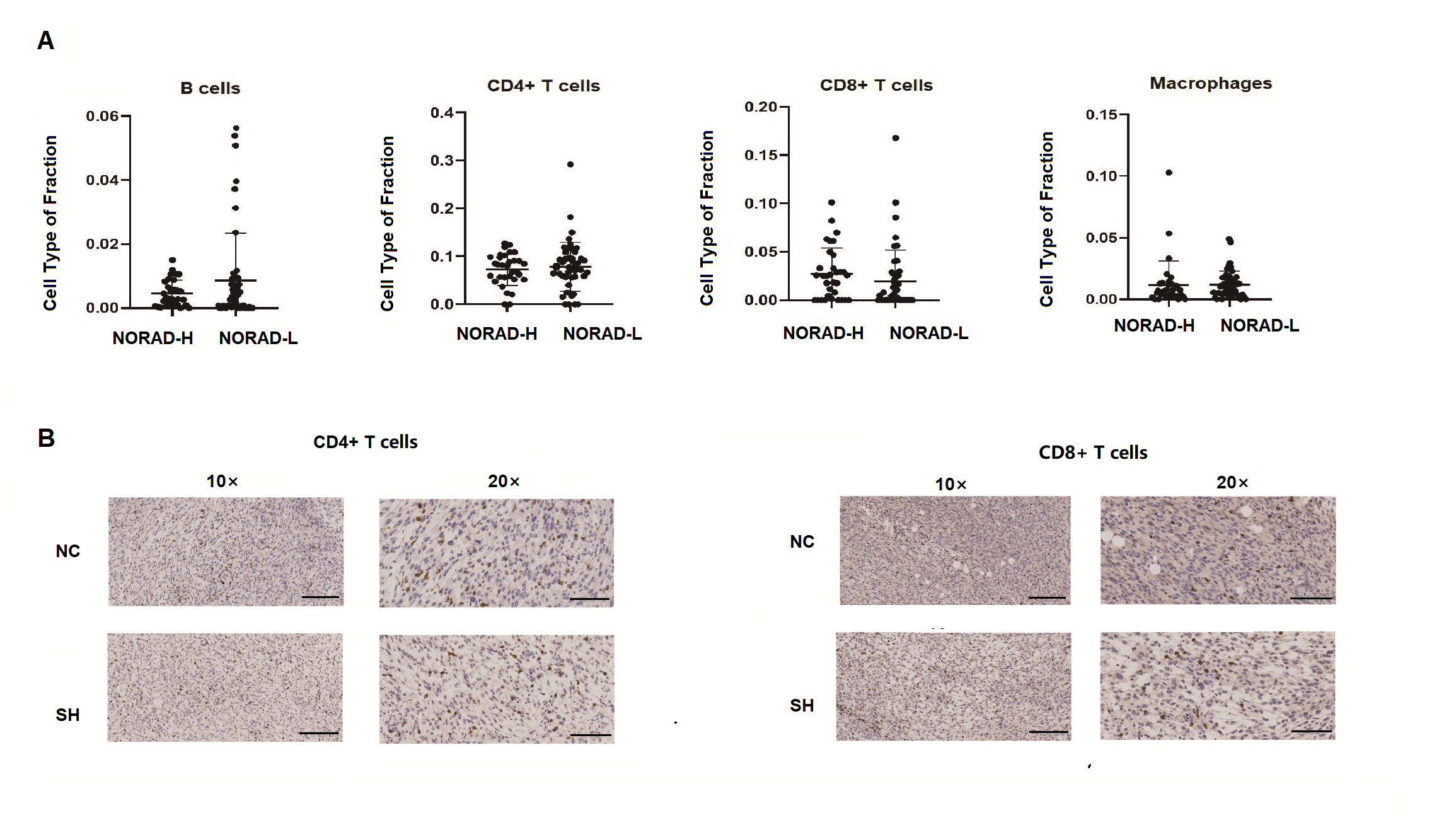

Supplement: Supplementary file 6 — Additional file 6: Supplementary Fig. 6. A. CIBERSORT results of the fraction of infiltrating immune cells (B cells, CD4+ T cells, CD8+ T cells and macrophages) in groups with high NORAD expression and low NORAD expression. The two groups were separated according to the mean value of NORAD expression. Data were extracted from TCGA datasets. B. Representative images of IHC staining for CD8+ T cells and CD4+ T cells in SH-NC-AKR and SH-NORAD-AKR cell-derived tumours. The scale bar in IHC images (10×) represents 200 μm, and the scale bar in other images (20×) represents 100 μm. [file 13046_2021_2084_MOESM6_ESM.tiff]
