# Supplementary material for: Radiation induces NORAD expression to promote ESCC radiotherapy resistance via EEPD1/ATR/Chk1 signalling and by inhibiting pri-miR-199a1 processing and the exosomal transfer of miR-199a-5p
Source: J Exp Clin Cancer Res. 2021 Sep 29;40:306. doi: 10.1186/s13046-021-02084-5 (PMC8479908; doi:10.1186/s13046-021-02084-5)
Supplement: Supplementary file 7 — Additional file 7: Table S1. Clinical characteristics of ESCC patients in radio-resistant group and the radio-sensitive group in TCGA datasets. [file 13046_2021_2084_MOESM7_ESM.docx]

|  |  | Sensitive | Resistant | P value |
| --- | --- | --- | --- | --- |
| Gender | Female | 3 | 0 |  |
|  | Male | 22 | 9 | 0.549 |
| Age | <60 | 13 | 4 |  |
|  | ≥60 | 12 | 5 | 1.000 |
| Somking | non-smoker | 10 | 2 |  |
|  | somker | 15 | 7 | 0.582 |
| AJCC | I-II | 19 | 7 |  |
|  | III-IV | 6 | 2 | 1.000 |
| T stage | T1-T2 | 14 | 2 |  |
|  | T3-T4 | 11 | 7 | 0.177 |
| N satge | N0 | 17 | 6 |  |
|  | N1 | 8 | 3 | 1.000 |
| M stage | M0 | 24 | 7 |  |
|  | M1 | 1 | 2 | 0.164 |
| Location | Proximal | 13 | 6 | 0.854 |
|  | Distal | 9 | 3 |  |
|  | Mid | 3 | 0 |  |

Table1 Clinical characteristics of ESCC patients in radio-resistant group and the radio-sensitive group in TCGA datasets.
