# Supplementary material for: Radiation induces NORAD expression to promote ESCC radiotherapy resistance via EEPD1/ATR/Chk1 signalling and by inhibiting pri-miR-199a1 processing and the exosomal transfer of miR-199a-5p
Source: J Exp Clin Cancer Res. 2021 Sep 29;40:306. doi: 10.1186/s13046-021-02084-5 (PMC8479908; doi:10.1186/s13046-021-02084-5)
Supplement: Supplementary file 8 — Additional file 8: Table S2. Clinical characteristics of 77 ESCC patients in chemoradiotherapy resistant group and the chemoradiotherapy sensitive group. [file 13046_2021_2084_MOESM8_ESM.docx]

|  |  | Sensitive | Resistant | P value |
| --- | --- | --- | --- | --- |
| Gender | Female | 18 | 8 |  |
|  | Male | 30 | 21 | 0.520 |
| Age | <60 | 24 | 12 |  |
|  | ≥60 | 24 | 17 | 0.618 |
| Somking | non-smoker | 28 | 14 |  |
|  | somker | 20 | 15 | 0.533 |
| AJCC | I-II | 32 | 16 |  |
|  | III-IV | 16 | 13 | 0.444 |
| T stage | T1-T2 | 12 | 11 |  |
|  | T3-T4 | 36 | 18 | 0.345 |
| N satge | N0 | 33 | 16 |  |
|  | N1-3 | 15 | 13 | 0.334 |
| M stage | M0 | 38 | 25 |  |
|  | M1 | 10 | 4 | 0.637 |
| Location | Proximal | 15 | 6 |  |
|  | Distal | 29 | 21 |  |
|  | Mid | 3 | 2 | 0.566 |

Table1 Clinical characteristics of 77 ESCC patients in chemoradiotherapy resistant group and the chemoradiotherapy sensitive group.
