# Supplementary material for: Radiation induces NORAD expression to promote ESCC radiotherapy resistance via EEPD1/ATR/Chk1 signalling and by inhibiting pri-miR-199a1 processing and the exosomal transfer of miR-199a-5p
Source: J Exp Clin Cancer Res. 2021 Sep 29;40:306. doi: 10.1186/s13046-021-02084-5 (PMC8479908; doi:10.1186/s13046-021-02084-5)
Supplement: Supplementary file 9 — Additional file 9: Table S3. The correlation between EEPD1 expression and radiotherapy outcome in 77 ESCC patients. [file 13046_2021_2084_MOESM9_ESM.docx]

|  | Radio-resistance | Radio-sensitive | Total | χ^2^ | P value |
| --- | --- | --- | --- | --- | --- |
| High expression | 20 | 17 | 53 | 8.151 | 0.004** |
| Low expression | 9 | 31 | 14 |  |  |
| Total | 29 | 48 | 77 |  |  |

Table 1. The correlation between EEPD1 expression and radiotherapy outcome in 77 ESCC patients.
